# Exosome nanovesicles as potential biomarkers and immune checkpoint signaling modulators in lung cancer microenvironment: recent advances and emerging concepts

**DOI:** 10.1186/s13046-023-02753-7

**Published:** 2023-08-29

**Authors:** Naushad Ahmad Khan, Mohammad Asim, Kabir H. Biswas, Amani N Alansari, Harman Saman, Mohammad Zahid Sarwar, Kudaibergen Osmonaliev, Shahab Uddin

**Affiliations:** 1 Department of Surgery, Trauma and Vascular Surgery Clinical Research, Hamad General Hospital, 3050 Doha, Qatar; 2 Faculty of Medical Sciences, Ala-Too International University, Bishkek, Kyrgyzstan; 3 Division of Biological and Biomedical Sciences, College of Health & Life Sciences, Hamad Bin Khalifa University, Qatar Foundation, Doha, Qatar; 4 Department of Medicine, Hazm Maubrairek Hospital, Al-Rayyan, Doha, 3050 Qatar; 5 Translational Research Institute & Dermatology Institute, Academic Health System, Hamad Medical Corporation, Doha, 3050 Qatar; 6 Department of Biosciences, Integral University, Lucknow, 226026 UP India

**Keywords:** Tumor-derived exosomes, Lung cancer, Biomarkers, Immune checkpoint signaling inhibitors, Tumor micro-environment, Immunotherapy

## Abstract

Lung cancer remains the leading cause of cancer-related deaths globally, and the survival rate remains low despite advances in diagnosis and treatment. The progression of lung cancer is a multifaceted and dynamic phenomenon that encompasses interplays among cancerous cells and their microenvironment, which incorporates immune cells. Exosomes, which are small membrane-bound vesicles, are released by numerous cell types in normal and stressful situations to allow communication between cells. Tumor-derived exosomes (TEXs) possess diverse neo-antigens and cargoes such as proteins, RNA, and DNA and have a unique molecular makeup reflecting tumor genetic complexity. TEXs contain both immunosuppressive and immunostimulatory factors and may play a role in immunomodulation by influencing innate and adaptive immune components. Moreover, they transmit signals that contribute to the progression of lung cancer by promoting metastasis, epithelial-mesenchymal transition (EMT), angiogenesis, and immunosuppression. This makes them a valuable resource for investigating the immune environment of tumors, which could pave the way for the development of non-invasive biomarkers that could aid in the prognosis, diagnosis, and immunotherapy of lung cancer. While immune checkpoint inhibitor (ICI) immunotherapy has shown promising results in treating initial-stage cancers, most patients eventually develop adaptive resistance over time. Emerging evidence demonstrates that TEXs could serve as a prognostic biomarker for immunotherapeutic response and have a significant impact on both systemic immune suppression and tumor advancement. Therefore, understanding TEXs and their role in lung cancer tumorigenesis and their response to immunotherapies is an exciting research area and needs further investigation. This review highlights the role of TEXs as key contributors to the advancement of lung cancer and their clinical significance in lung immune-oncology, including their possible use as biomarkers for monitoring disease progression and prognosis, as well as emerging shreds of evidence regarding the possibility of using exosomes as targets to improve lung cancer therapy.

## Introduction

Lung cancer continues to be one of the most prevalent forms of malignant tumors, primarily bronchogenic carcinoma and is responsible for a disproportionately high percentage of cancer-related deaths worldwide [1]. Lung cancer can be classified into two histological types, namely small-cell lung cancer (SCLC) and non-small cell lung cancer (NSCLC), which make up 15% and 85% of all cases, respectively. Lung cancer is defined by different genetic alterations and a strong hereditary component [2], and the majority of patients are diagnosed at an advanced or metastatic stage (up to 85%), which is associated with a poor prognosis [3]. As a result, it is critical to study carcinogenesis and cancer therapies, especially for lung cancer, which would benefit greatly from early detection because delayed detection raises the risk of mortality [4, 5].

Recent advances in immunotherapy have dramatically altered the treatment perspective for lung cancer, with effectiveness rates far exceeding those of conventional chemotherapy [5–7]. Despite the limited success of immunotherapies in treating lung cancer, their effectiveness is restricted to a small subset of patients, and the development of primary and secondary resistance makes therapy more challenging [8]. One plausible justification for this phenomenon is the diverse composition of lung cancer cells, each with unique molecular and epigenetic alterations, resulting in a heterogeneous and complex tumor microenvironment (TME) [9, 10]. Furthermore, immunotherapy and medications that act as immune checkpoint inhibitors (ICIs) are only beneficial to a subset of patients with lung cancer [8, 11, 12]. Therefore, advancing knowledge of molecular pathways underlying lung cancer, early identification and targeted therapeutic development are imperative.

Exosomes, first identified in the early 1980s as vesicles that slough off from both normal and cancerous cell lines, belong to the extracellular vesicle (EVs) family [13, 14]. Although previously considered cellular trash receptacles, they are now acknowledged for their ability to promote cross-talk with cellular surroundings [15, 16]. They are bioactive lipid bilayer nanovesicles with a 40–150 nm diameter released by practically all types of normal and malignant cells [16, 17] and are generated through the endo-lysosomal pathway and stem from a specific endosomal compartment called multivesicular bodies (MVBs) [18, 19]. Exosomes transport a variety of substances, mostly proteins, lipids, DNA and RNA (mRNA and non-coding RNA), crucial for intercellular signal transmission [15, 18].

Exosomes have a crucial function in the TME and can act as a diagnostic and prognostic marker for the alteration of TME and the progression of cancer to a certain degree [20]. Their importance stems from their ability to enable intercellular communication and convey a diverse range of micro-molecules and signaling chemicals between cancer cells and the adjacent cells that comprise the TME [17, 21]. Exosomes may promote tumor progression [22–25] by enhancing tumor cell proliferation [26–29], angiogenesis [30–33] and metastasis [21, 34].

Exosomes, as major carriers of cell content exchanges, have received much interest for their function in lung cancer [34–41] and other forms of cancer [42–49], as well as inhibiting immunological responses and regulating TME [37, 50, 51]. With physiologically active proteins, they can decrease cytotoxicity and modulate the expression of genes associated with immunity in T cells, thus augmenting the capacity of tumor cells to evade the immune system [51]. Furthermore, normal and cancerous cells exhibit significant differences in the amount and composition, indicating a certain degree of selectivity [52]. As a result, the identification of exosomes can be beneficial in disease diagnosis and tumor prognosis. Based on findings of a positive correlation between tumor-derived exosomes (TEXs) with attenuated immunity and responses from immunotherapy, the role of exosomes in tumor immunity and immunotherapy response is an area of current research. TEXs have been associated with immunological intercommunication, signaling and are believed to be a promising biomarker for lung cancer immunotherapy [20, 37, 50, 53, 54], and an intriguing aspect is their release by cancer cells and ability to affect normal cells. Moreover, they have potential utility as a diagnostic tool for various cancers as they are found in diverse biological specimens such as blood, urine, cerebrospinal fluid and saliva [14, 41]. Further, due to their ability to convey signals between tumor and immune cells, TEXs are targeted in novel cancer immunotherapy developments [55–57].

Although the advent of immune checkpoint inhibition therapy has significantly changed the cancer treatment landscape [58], to fully comprehend this therapy's efficacy, a deeper understanding of its successes and failures is crucial. Recent investigations into the composition of exosomes have uncovered the presence of various immune checkpoint proteins in them, particularly those that originate from tumors, such as programmed death ligand 1 (PD-L1) [59]. Many researchers now hypothesize that immune checkpoint proteins in exosomes play a crucial role in a new mechanism for mediating tumor immune evasion. This suggests that targeting these checkpoint molecules could represent a novel approach to cancer immunotherapy, with immune checkpoint blockade as a promising method for activating anti-tumor immunity [58–64]. Such blocking of exosome secretion along with immune checkpoint proteins may further enhance the effectiveness of anti-tumor immune responses, paving the way for new possibilities in tumor immunotherapy.

Recently, exosomes have drawn much attention because of their potential to assist cancer patients with diagnostic and treatment outcomes using liquid biopsy [55, 65]. The nucleic acids, proteins, and lipids found in TEXs may also share traits with their parental cells, making them a possible source of new biomarkers [41, 55, 66]. Additionally, growing evidence suggests TEXs which freely circulate in bodily fluids and contain micro and long non-coding RNAs (miRNAs and lncRNAs), can be used as prognostic or predictive biomarkers for response to anti-tumor therapies in NSCLC [2, 40, 51, 66–68].

The current overview starts by describing how TEXs are produced and released into the extracellular matrix of cells and discussing the components that constitute TEXs. The review then delves into how TEXs can modulate the immune system, immunotherapy as a drug delivery vehicle, various immunological checkpoints and the potential for TEXs to serve as diagnostic and prognostic biomarkers for lung cancer, including TEX-derived cancer vaccines.

## Biogenesis and composition of tumor-derived exosomes (TEXs)

Exosome biogenesis is a well-regulated process influenced by lipid complexes and proteins involved in endocytosis [69] and initiated by the synthesis of early endosomes by engulfing specific domains of the cellular membrane. When the restricting membrane of initial sorting endosomes folds inwards, multiple intraluminal vesicles are formed, creating multivesicular bodies. Exosomes are ultimately discharged into the extracellular milieu by merging multivesicular structures with the plasma membrane [15, 38, 69].

Exosome production is a highly regulated process occurring through either of two mechanisms, one dependent on and another independent of the endosomal sorting complex required for transport (ESCRT) [69, 70]. ESCRT is well acknowledged as the primary regulator of early endosome development and late endosome transformation inside multivesicular bodies (MVBs) [71]. The other mechanism involves ceramide/tetraspanin-dependent pathway [72, 73].

Both ESCRT-dependent and independent pathways, may differ based on the type of cell and payloads. Moreover, additional signals and pathogenic stimuli the cell is exposed to might also have an impact. Exosomes, upon release, can transfer crucial information to their target cells through a variety of mechanisms. These mechanisms primarily include endocytosis or phagocytosis, and other processes (membrane fusion, micropinocytosis, Receptor- and Raft-mediated Endocytosis) whereby the exosomes are engulfed by the recipient cells and their contents are subsequently released within the cytoplasm [73]. Furthermore, receptor-ligand interactions between exosomes and target cells can trigger particular signaling pathways in the target cells, causing functional changes [71, 73]. This cargo delivery has substantial biological consequences ranging from changes in the transcriptome and proteome of the receiving cells to changes in their cellular activities [74]. Importantly, there is a possibility that certain exosomes are discharged from cells through a process of direct outward budding and fission of the plasma membrane, which is similar to the release of microvesicles and apoptotic blebs [73].

**Fig. 1:**
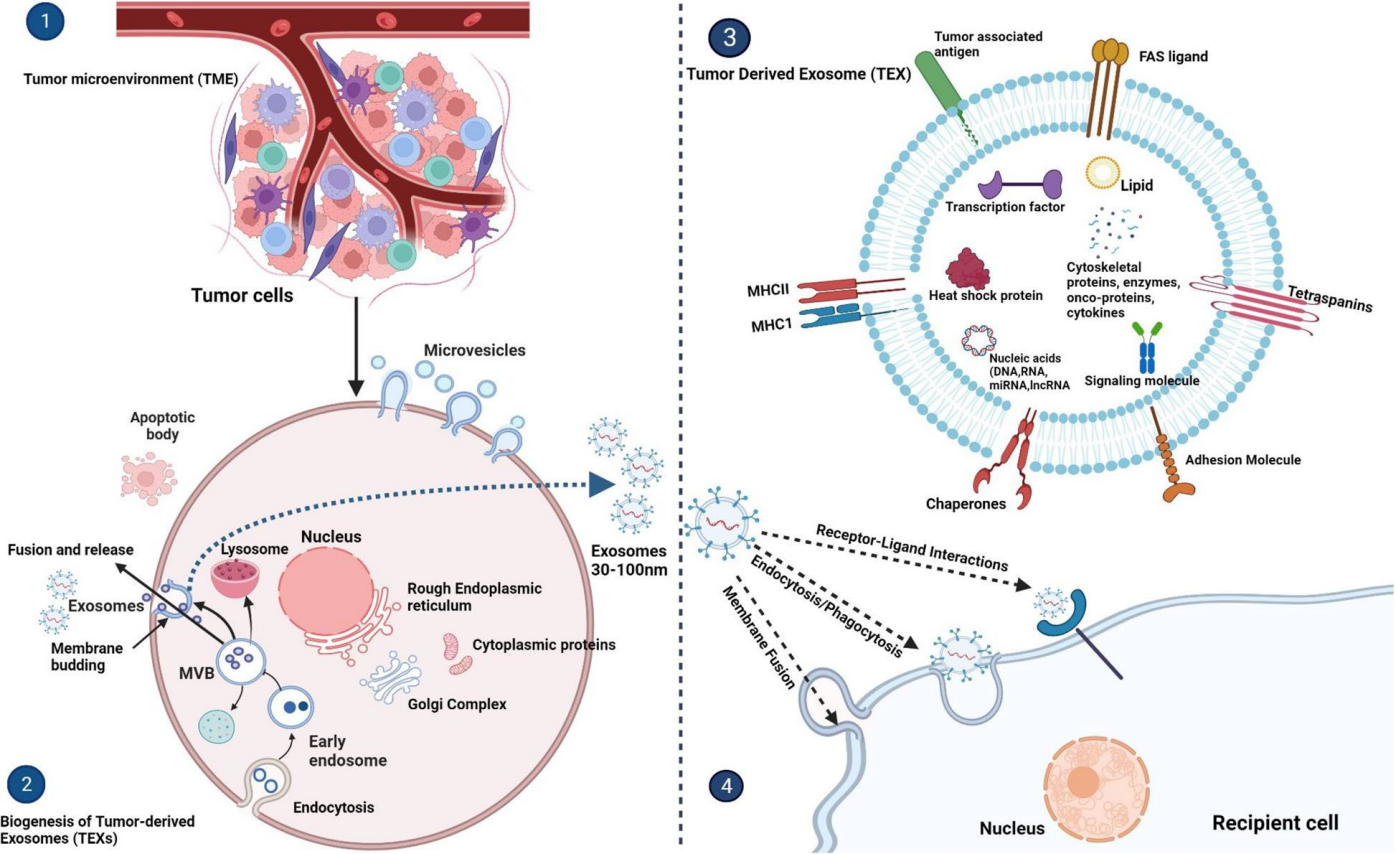
Biogenesis, molecular and cellular composition uptake and transfer mechanisms of tumor-derived exosomes (TEXs) in tumor microenvironment (TME): 1. the tumor microenvironment (TME) is contains tumor cells that coexist alongside immune cells, fibroblasts, and blood vessels, collectively forming the complex TME. 2. Tumor cells contain tumor-derived exosomes (TEXs) that are created from intraluminal vesicles within multivesicular bodies (MVBs). The process begins with the formation of early endosomes through the inward budding of membrane tiny domains from the plasma membrane. These endosomes, which ultimately become MVBs, are dynamic subcellular structures where RNA and cytoplasmic proteins are stored. These MVBs are either incorporated into exosomes by combining with the plasma membrane or they are degraded by lysosomes. Exosomes acquire new components during this transformation, including proteins, nucleic acids, and lipids. 3. Schematic representation of the cellular and molecular composition of TEXs. Specific payloads packed inside TEXs include a variety of proteins, DNAs, metabolites, lipids, mRNAs, and miRNAs, and lncRNAs. Specific membrane proteins that act as biomarkers, such as chaperones, tetraspanins, MHC I and II, tumor-associated antigens, growth factor receptors, and some cytoskeletal proteins, enzymes, are present on the surface of exosomes. 4. The exosomes ultimately release their contents, such as DNA, microRNA and proteins, into recipient cells via different mechanisms including endocytosis/phagocytosis, direct membrane fusion and receptor-ligand interactions. Figure created with BioRender.com

The lipid-protein bilayer membrane of TEXs present in all tumor tissues and bodily fluids contains a variety of proteins, including transport and fusion proteins, adhesion molecules, inhibitory ligands, MHC molecules, tetraspanins, tumor-associated antigens, chaperones, glycolipids [22, 38, 52, 69, 70, 75]. The intravesicular contents of TEXs and made up of proteins, lipids, nucleic acids, including mRNA, miRNA, long non-coding RNA, circRNA and are functional when taken up by target cells [37, 53, 74] (Fig. 1). Inside the TME, TEXs are able to transmit information from the primary tumor to the recipient cells, such as immune cells [50]. Therefore, TEXs carry oncogenic material to their destination cells which can have a wide range of effects depending on the cells and circumstances and are regarded as surrogates of parent tumor cells since they mirror the molecular and genetic makeup of these cells.

## TEXs and the tumor microenvironment in lung cancer

Cancer cells are aberrant cells that develop rapidly and uncontrollably, eventually producing abnormal tissue and tumors. The development and advancement of tumors rely significantly on the characteristics of the surrounding microenvironment, which includes both cellular and non-cellular factors [37, 76]. Exosomes play a vital role in transmitting messages between cells and regulating the TME, which is composed of diverse components exhibiting different characteristics depending on the origin of the tumor [20, 76]. The most predominant components of TME include carcinoma cells, immune cells (such as T and B lymphocytes, natural killer cells, dendritic cells, and macrophages), extracellular matrix (ECM), stromal cells (including fibroblasts and adipocytes), a system of lymphatic and blood vessels [77]. The molecular and cellular characteristics of the TME influence the local immune responses, which consequently affects the severity of the malignancy [78]. Furthermore, TME cells communicate directly and indirectly through cell-to-cell interaction and the production of soluble substances and vesicles, and direct cell contact and signaling are facilitated by cell junctions and ligand-receptor recognition. Thus, with exosomes acting as key communication molecules between cancer cells and adjacent cells, TME is vital in carcinogenesis, progression, and treatment response [37, 50].

**Fig. 2:**
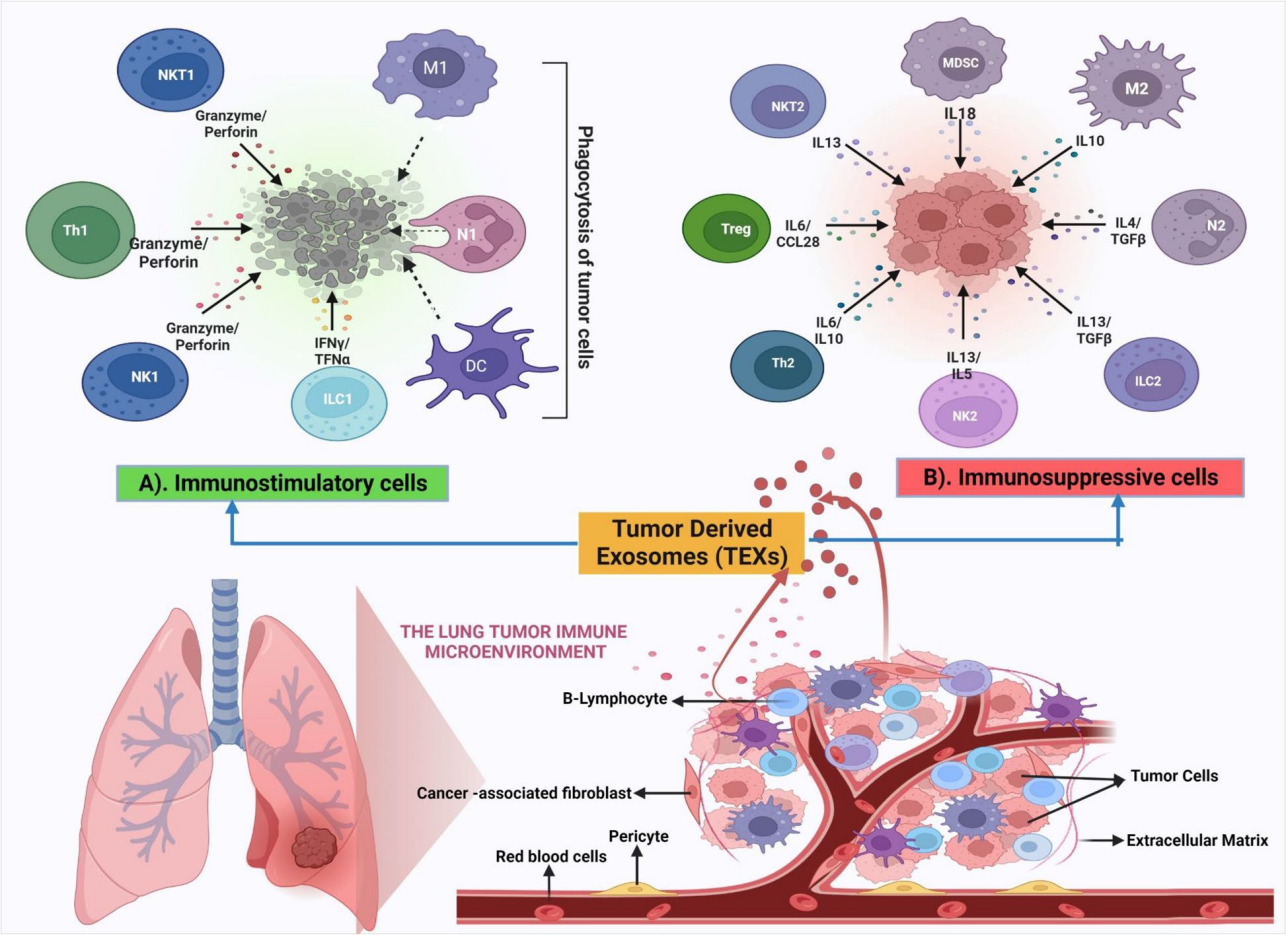
Tumor-derived exosome (TEX) mediated communication and immunomodulation in the tumor microenvironment (TEX): TEXs transmit immunosuppressive and immunostimulatory signals to immune cells, influencing the immunological response in TME. In terms of immunological suppression, TEXs can cause immune cells like T cells to undergo apoptosis which is required for an efficient immune response against cancer cells. Moreover, TEXs can limit the function of effector T lymphocytes impairing tumor cell killing. TEXs have also been demonstrated to increase macrophage M2 polarization, promoting tumour development and blocking the immunological response to tumors. TEXs can also increase the number of MDSCs, immunological cells that reduce T cell function, further suppressing the anti-tumor immune response. TEXs have also been reported to decrease the development of dendritic cells required for T cell activation and initiating an immunological response. However, TEXs can stimulate the immune system, boost anti-tumor activity and increase the activity of macrophages and NK cells, which play crucial role in destroying cancer cells. TEXs have also been shown to block macrophage M2 polarization which can stimulate an anti-tumor immune response and directly or indirectly boost T cell activation resulting in greater tumor cell death. These immunostimulatory activities of TEXs have the potential to stimulate an effective immune response against cancer and perhaps enhance cancer patient outcomes. Figure created with BioRender.com

Exosomes that originate from tumor cells are commonly known as TEXs [79]. The nucleic acids present in TEXs, such as microRNAs and messenger RNAs, can be transferred from the tumor cells to other cells in the microenvironment, where they can influence gene expression and cellular behavior. When transferred to recipient cells, they carry both activating and inhibiting components that facilitate communication between tumor cells and their microenvironment. In addition, exosomes are known to participate in various biological processes such as regulating the immune response, controlling EMT [80], influencing the function of cancer-associated fibroblasts (CAFs) [81], and engaging in a crucial role in angiogenesis [32]. TEX-mediated communication and immunomodulation in the tumor microenvironment are illustrated in Fig. 2.

## Tumor-derived exosomes (TEXs) and immune response modulation in lung cancer

In the course of cancer development, cellular crosstalk in the TME and various immune cells is a central driver of tumor progression. When it comes to cancer, the immune system has a double-edged sword effect in encouraging chronic inflammation and stifling antitumor immunity while also killing cancer cells and slowing tumor development. Recent research suggests TEXs play a vital role as important mediators of cellular communication by transmitting both immuno-inhibitory and immune-stimulatory signals to immune cells of TMEs in lung cancer. The effect of immune cell-derived TXEs on the TME in lung cancer is outlined is Table 1 in accord with the recent studies.

**Table 1:** Recent studies on the effect of immune cell-derived exosomes (TXEs) on the tumor microenvironment (TME) in lung cancer

Immune cells	Cellular source/Molecule	Isolation method	Comments	References
Tumor-Associated Macrophages (TAMs)	A549 cells/Vesicular cargoes	Ultracentrifugation	TEXs derived from lung tumors are capable of inducing a shift in the phenotype of M0 macrophages towards the M2 type by modifying their transcriptional and bioenergetic profiles	[82]
Tumor-Associated Macrophages (TAMs)	NCI-H1792, NCI-H1437, NCI-H2087 (Human adenocarcinoma cell lines) *miR-103a*	Exosome isolation kits (Exo-ELISA)	Lung cancer-derived exosomes exposed to hypoxia boosted M2-type polarization via *miR-103a* transfer, which upregulated AKT and STAT3 activation and the expression of multiple immunosuppressive and pro-angiogenic markers	[83]
Tumor-Associated Macrophages (TAMs)	A549 & H23 cells *miR-21-5p*	Precipitation, TEM	Exosomes originating from HMSCs, which were exposed to hypoxia prior, substantially boosted the growth, endurance, invasion, EMT, and increased the expression of miR-21-5p. This, in turn, suppressed apoptosis and increased M2 polarization of macrophages, leading to the promotion of lung cancer development	[84]
Tumor-Associated Macrophages (TAMs)	Adenocarcinoma human alveolar basal epithelial cells (A549) / Vesicular cargoes	Precipitation, TEM	Exosomes from hypoxic A549 cells increased proliferation, migration, EMT, and PKM2 expression leading to PKM2-induced M2 polarization of macrophages through the AMPK pathway resulting in tumor development, and lung metastasis	[85]
Tumor-Associated Macrophages (TAMs)	A549 cells / Vesicular cargoes		The invasion and metastasis of A549 cells are facilitated by miR-10b in exosomes released by tumor cells, which induces polarization of M2 macrophages	[86]
Cancer-associated fibroblasts (CAFs)	CAFs from NSCLC cancer tissue and NFs from para-carcinoma tissue/ *miR-103a-3p*	Ultra-centrifugation, TEM	CAFs produce exosomal miR-103a-3p that hinders apoptosis by targeting Bak1, which could be a promising therapeutic option for addressing cisplatin resistance in NSCLC	[87]
Cancer-associated fibroblasts (CAFs)	A549 cells PBMC cells		Exosomal OIP5-AS1 from CAFs inhibited PBMC-induced cell apoptosis and enhanced lung cancer proliferation by decreasing *miR-142-5p* and increasing PD-L1	[88]
Cancer-associated fibroblasts (CAFs)	Embryonic mouse fibroblast cell line (NIH3T3) / *miR-210*	Ultra-centrifugation, TEM	Exosomes generated from LCs may stimulate cell reprogramming, allowing fibroblasts to differentiate into CAFS, and overexpression of exosomal miR-210 might promote angiogenesis and may trigger the expression of proangiogenic proteins via the JAK2/STAT3 signaling pathway	[89]
Myeloid-derived suppressor cells (MDSC)	95D (human lung cancer cells), H292 (lung mucoepidermoid carcinoma cells), H358 ( lung adenocarcinoma cells), *miR-21a*	Ultra-centrifugation, TEM	Exosomes from lung carcinoma cells contain miR-21a which targets PDCD4 to promote the proliferation of MDSCs, accelerating tumor progression	[90]
Myeloid-derived suppressor cells (MDSC)	Human lung cancer cell lines, and mouse lung cancer cell line, Lewis LLC cell line /*miR-143-3p*	Ultracentrifugation and TEM	More exosomes were produced by G-MDSCs in lung cancer tissues, thereby promoting cancer progression. Upregulation of *miR-143-3p* in G-MDSCs-derived exosomes significantly attenuates ITM2B by binding to its 3'-untranslated region	[91]
Dendritic Cells (DCs)	LLC, A549, and Beas-2b/ MALAT1	Precipitation, (exosome extraction kit)	MALAT1 inhibition may be a promising method for treating lung cancer since it increases DC function and T cell proliferation while suppressing DC autophagy and T proliferation and differentiation in LLC-derived exosomes when MALAT1 expression is inhibited	[92]
Dendritic Cells (DCs)	A549 cells LLC cells	Ultracentrifugation	TEXs effectively promote DC maturation and increase MHC cross-presentation, leading to a more potent tumor-specific cytotoxic T-cell response. More crucially, TEXs lower DC PD-L1 expression resulting in a down-regulated population of Tregs in vitro	[93]
Dendritic Cells (DCs)	BMDCs/EGFR gene mutation E746-A750	Ultracentrifugation	Loss of intratumoral CD8 + T lymphocytes correlates with the progression of harbouring the E746-A750 deletion mutation (EGFR-19del). Early-stage EGFR-19del-expressing LLC tumors are characterized by a low T cell density and the presence of DC with diverse morphologies	[94]
T cells	A549, PC9,95D/CircRNA-002178	Exosome Precipitation Solution kit	CircRNA-002178 might increase PD-L1 expression in cancer cells by syphoning miR-34, causing T-cell fatigue, and could serve as biomarkers for early detection of lung adenocarcinoma	[95]
T cells	Human NSCLC cell lines	Ultracentrifugation	Exosomes generated from lung cancer cells express PD-L1, which aids immunological evasion by decreasing T cell activation and encouraging tumor development. Exosomes can reduce cytokine production and induce death in CD8 + T cells, impairing immunological activity	[96]
T cells	Human NSCLC cell lines	ExoQuick Exosome Precipitation Solution kit	NSCLC cells are responsible for the majority of exosomal circUSP7 secretion, and this leads to immunosuppression in NSCLC by increasing malfunction in CD8 + T cells	[97]
Natural killer cells (NKs)	A549, Mouse NK cell line (LNK)	ExoEasy Maxi kit precipitation lit	Modulating the expression of miR-30c could be a potential strategy to enhance NK cell-based anticancer treatments. This is suggested by its ability to increase the cytotoxicity of NK cells towards lung cancer cells through the reduction of GALNT7 and inactivation of the PI3K/AKT pathway	[98]

*Abbreviations***:** *A549* Adenocarcinoma human alveolar basal epithelial cells, *BMDCs* Bone Marrow-Derived Dendritic Cell, *circUSP7* circular ubiquitin-specific protease-7, *DC* Dendritic cells, *GALNT7* polypeptide-N-acetyl-galactosaminlytransferase 7, *G-MDSCs* Granulocytic myeloid-derived suppressor cells, *EGFR* Epidermal Growth factor Receptor, *ITM2B* integral membrane protein 2B, *LLC* Lewis lung carcinoma, *MALAT1* Lung adenocarcinoma metastasis-associated transcript 1, *NFs* Normal fibroblasts, *NSCLC* Non small cell lung carcinoma, *PD-L1* Programmed death ligand-1, *TEM* Transmission electron microscopy, *Tregs* T regulatory cells

## Natural killer cells (NKs) and interaction between exosomes in the lung TME

Natural killer (NK) cells are components of innate immune cells capable of acting independently and expressing several receptors with stimulatory or suppressive functions [35]. They can directly attack tumor cells without being restricted by a major histocompatibility complex (MHC) and play a crucial role in immuno-surveillance [99]. Tumor cells, on the other hand, interfere with normal NK cell activities and impede cytotoxicity in the TME [77]. In lung cancer, the degree of NK cell infiltration is associated with survival rate [20]. Recent research has shown that in lung cancer, TEXs containing miR-21/29a can attach to internal toll-like receptors (TLRs) located on natural killer cells (NKs). This attachment activates a protein called NF-B, leading to the initiation of an inflammatory response that supports metastasis and the advancement of the tumor [100].

Additionally, hypoxia also impairs the immune characteristics of NK cells [101]. The reduced activity of NK cells, which has been observed in lung cancer patients, is likely a result of the suppression of cell-surface receptors, specifically the natural killer group 2D (NKG2D) receptor, a member of a family of transmembrane proteins that function as activators and resemble C-type lectins [101, 102]. Transforming growth factor β1(TGF-β1) is transferred to NK cells via exosomes generated from hypoxic tumor cells, which decrease NK cell activity, leading to desensitization and inhibition of NKG2D receptors in lung cancer [101, 103]. In hypoxic exosomes, miR-23a functions as an immune-inhibitory component by overwhelming the expression of CD107a in NK cells [101]. Notably, in NK cells, endocytosis of NKG2D suppresses the production of surface receptors and controls how cells respond to signals. TEXs are implicated in the inhibition of NK cell function through several mechanisms, including the dampening of IL-2 signaling pathways, decreased synthesis of perforin, and the activation of Janus kinase (JAK-3) [104].

## Dendritic cells (DCs) regulation by TEXs in lung TME

Dendritic cells (DCs) are natural immune cells that play an important role in coordinating the immune system's reaction to malignant tumors. These cells are derived from hematopoietic progenitor cells and serve an important part in the natural immunological response [105]. DCs act as antigen-presenting cells (APCs) and identify and ingest pathogens before displaying them to immune cells such as T cells. This process involves the interaction of surface receptors and co-stimulatory proteins, which activate the immune response [106]. Furthermore, DCs release cytokines and chemokines, which can influence the microenvironment and tumor formation [106, 107]. TEXs have been implicated in immune system modulation by influencing monocyte differentiation and maturation, and TMEs are well known for instructing DCs to enhance tumorigenicity [108]. Exosomes released by lung tumor cells play a crucial role in this scenario by efficiently transporting a wide range of tumor antigens to DCs as well as shuttling signaling molecules and facilitating cell-to-cell contact [109]. TEXs associated with lung cancer have been found to be highly efficient at delivering various tumor antigens to DCs and are observed to stimulate DC maturation and MHC cross-presentation resulting in a specific cytotoxic T cell response against tumors [93, 110].

Huang et al. have demonstrated how exosomes derived from lung cancer biopsies possess heightened levels of epidermal growth factor receptor (EGFR) [111]. These exosomes can activate tolerogenic dendritic cells (DCs) and regulatory T cells, resulting in the reduced expression of tumor antigen-specific CD80 and CD86 cells leading to the inhibition of DC maturation and function [111]. Furthermore, Li et al. demonstrated that DC exosomes carrying neoantigens could decrease tumor development, prolong survival, inhibit tumor formation and eradicate lung metastasis. Dendritic cell-derived nano-vaccines induce significant immune responses from antigen-specific and broad-spectrum T and B cells [112]. The treatment is safe and compatible, suggesting that it may have the potential for personalized immunotherapy in lung cancer.

## Myeloid-derived suppressor cells (MDSCs) regulation by TEXs in lung TME

The TME contains a heterogenous group of immature myeloid cells known as myeloid-derived suppressor cells (MDSCs), which possess immunosuppressive properties [113] and whose activation and proliferation through intercellular communication are aided by tumor cells in the TME. Once expanded, MDSCs suppress the immune system's response against the tumor. Their behaviour can be altered by TEXs, whose production and release can be influenced by the TME [65]. This includes facilitating their activation, promoting their growth and enhancing their ability to suppress the immune system. Cancer is associated with an increase in MDSCs, an immature population of myeloid cells known to inhibit the antitumor T cell response [114]. MDSCs normally shield the body from the detrimental effects of excessive immune reactions during abnormal events such as wound repair.

Nevertheless, they promote angiogenesis, invasion, and metastasis while suppressing anti-tumor activity within the tumor microenvironment [115]. It has been demonstrated that TEXs cause the differentiation of myeloid progenitor cells into MDSCs with potent suppressive characteristics. Furthermore, MDSCs control T cell activity to create an immunosuppressive milieu, and exosomes containing pro-inflammatory cytokines (interleukin-6 [IL-6], tumor necrosis factor-α [TNF-α] and tumor-associated cytokines (granulocyte–macrophage colony-stimulating factor [GM-CSF], macrophage colony-stimulating factor [M-CSF]) aid in this process [65]. Moreover, exosomes released from lung cancer cells specifically target a population of MDSCs as their primary target. T cell proliferation is inhibited by the internalization of lung cancer-derived exosomes by MDSCs which leads to an increase in the production of molecules such as TGFβ- and PGE2 [116].

Additionally, in vivo mice studies have demonstrated that TEXs trigger the accumulation of MDSCs and the heightened secretion of inflammatory mediators, such as IL-6 and vascular endothelial growth factor (VEGF), as well as immunosuppressive agents like nitric oxide and reactive oxygen species, ultimately leading to T cell death [117]. Available evidence also suggests that exosomal heat-shock protein 72 (Hsp72) located on the surface of TEXs can stimulate signal transduction and activation of transcription 3 (STAT3) [118]. This, in turn, triggers the production of autocrine IL-6 in MDSCs via toll-like receptor/MyD88 (TLR2/MyD88), further enhancing the immunosuppressive capacity of MDSCs [119].

## Tumor-associated macrophages (TAMS) regulation by TEXs in lung TME

The TME plays a pivotal role in the progression and spread of tumors. Macrophages are one of the immunological cells most prevalent in the TME and show distinctive phenotypic alterations in response to varying environmental conditions. These phenotypes can be broadly categorized into two types: M1 macrophages, which are classically activated, and M2 macrophages, which are alternatively activated. M1 macrophages have anti-tumor properties, while M2 macrophages possess anti-inflammatory characteristics and contribute to tumor promotion [120]. Tumor-associated macrophages (TAMs), which include both resident macrophages and monocytes drawn to the TME, are defined as macrophages that infiltrate the tumor tissues in the TME and are vital for fostering pre-metastatic niches, facilitating tumor progression, enabling chemoresistance, proliferation, metastasis, survival, and genetic instability of cells. TAMs exhibit an immunosuppressive behavior similar to M2-like macrophages [121]. The significance of TAMs in these pathways has been underlined in several research [122]. The two primary mechanisms underlying these activities are classic and alternative activation of macrophages. Unlike alternative activation (M2), which is associated with immunosuppression, cancer and angiogenesis, classical activation of macrophages (M1) is characterized by the production of antitumor and pro-inflammatory macrophages [123]. Exosomes secreted by NSCLC cells have the capacity to induce M0 macrophage and myeloid-derived suppressor cell development into M2 macrophages. Furthermore, exosome-induced M2 polarization is not dependent on the p53 gene [26]. TAM-derived exosomes have a unique proteomic profile and more active proteases. Further, exosomes induced by hypoxia increase macrophage recruitment and facilitate M2-like polarization in both in vitro and in vivo settings, and hypoxic lung cancer cell-derived exosomes polarize macrophages to the M2-type phenotype via miR-103a [83].

In monocyte survivability and TAM production within the tumor-inflammatory microenvironment, TEXs play a crucial role. Fibrosis, metabolism, cellular debris and T cell activity are directly and indirectly influenced by macrophages by the secretion of pro-inflammatory chemicals [122]. For example, STAT3 is largely phosphorylated by IL-6 generated by macrophages which promote tumor growth and metastasis [124]. It has been shown that Neuropilin-2 (NRP2), produced by TAMs, stimulates the formation of tumors by controlling the efferocytosis of tumor cells that have died [125]. Additionally, this pathway supports immunological suppression. Furthermore, it has been found that NSCLC cells can acquire features similar to cancer stem cells (CSC) when exposed to TAM-derived interleukin-10 (IL-10). The JAK1/STAT1/NF-B/Notch1 signaling pathway mediates this action [126].

Furthermore, the amount of pro-inflammatory cytokines in lung cancer enhancing carcinogenesis and metastasis can be boosted by TEXs, and the regulation of their release is significantly related to microRNAs [127]. The EV-carrying microRNAs produced from lung cancer miR-16, -21 and -29a bind to TLR7/8 on the surface of macrophages to provoke phosphorylation activation of NF-kB, which in turn, promotes an increase in the production of pro-inflammatory cytokines such as IL-6 [128].

## Effect of TEXs on Treg/T cell regulation in lung cancer

TEXs have a wide variety of pathways at their disposal to influence T cells. By lowering the stability, proliferation and regulatory activities of these, TEXs are able to change anti-tumor response [129]. They can also affect T cell effector function directly or indirectly by suppressing activated CD8 + T cell activity, causing CD8 + T cell death through pro-apoptotic molecules, stimulating Treg expansion and inducing T cell depletion [130]. Exosomes produced from tumors have been shown to be responsible for the transformation of CD4 + CD25neg T cells into CD4 + CD25highFOXP3 + T regulatory cells (Tregs) [81, 130]. Research has shown that the fraction of CD4 + CD25 + Foxp3 + Tregs in the TME and functional modifications of T lymphocyte subsets are essential for the immune evasion of lung cancer cells. The available data suggests lung cancer cell-derived exosomes affect DCs leading to an increase in the development of Treg cells in the TME, a reduction in the quantity of CD4 + T cells and inhibition of IFN-γ generation [130].

Furthermore, the adenosine pathway is regulated by TEXs, increasing CD39 expression and adenosine synthesis in Treg [131]. CD73 and CD39 are two enzymes found on Treg's surface that catalyse adenosine production from ATP [132]. TEXs have surface CD39 and CD73 and directly distribute membrane-tethered CD73 to CD39 + cells [132]. Further, they reduce local immunity by generating extracellular adenosine, which negatively regulates T-cell activation [54]. These TEX-mediated processes play a significant role in tumor resistance regulation and can increase tumor invasion in malignancies.

PD-L1 immunotherapy is increasingly used in clinical practice to target exosomal PD-L1, which modulates T-cell activity and the immune environment around cancerous tumors [133]. Recent studies indicate that tumor cells produce exosomal PD-L1, which hinders T-cell activation and promotes tumor growth [134]. It has been demonstrated that MiR-214 gets transported into recipient CD4 + T cells by TEXs to downregulate the PTEN-mediated signaling, promoting Treg proliferation and tumor progression [135], and it is interesting to note that co-incubation of Treg with TEXs may increase Treg quantity as well as its suppressive activity in lung cancer [136].

## Modulation of cancer-associated fibroblasts (CAFs) by TEXs in lung cancer

Fibroblasts are normally stimulated to aid in the healing of wounds by producing an extracellular matrix (ECM) that acts as a scaffold for other cells [137]. Myofibroblast-like cancer-associated fibroblasts (CAFs) commonly constitute the majority of the TME and can modulate fibroblast activity [138]. Contrary to typical fibroblasts, CAFs emit pro-invasive chemicals such as ECM-degrading proteolytic enzymes and produce excessive ECM. Therefore, they promote ECM restructuring and infiltration by producing a variety of cytokines, chemokines, matrix-degradable enzymes and growth factors [139]. Unknown molecular processes in the TME cause normal fibroblasts (NFs) to develop into CAFs and the phenotype of fibroblasts can be altered by exosomes and associated substances released by lung cancer cells [140]. Exosomes formed from CAF that include amino acids, lipids and intermediates of the TCA cycle deliver nutrients to malignant cells by a process analogous to micropinocytosis and are consumed by cancer cells for energy metabolism [141]. Therefore, exosomes enhance tumor development in situations that involve nutritional shortage or nutrient stress.

MiRNAs have a significant role in fibroblast differentiation and activation, as shown by the fact that dysregulation of miR-142-3p production causes normal fibroblasts to differentiate into CAFs by altering TGF-signaling [141]. It is also believed that CAF induces tumor angiogenesis, and some studies have reported that exosomes derived from lung cancer patients can modulate and induce cancer cell reprogramming. In lung cancer patients, exosomes overexpressing miR-210 can stimulate CAF activities and promote the synthesis of proangiogenic proteins via activating the JAK2/STAT3 pathway [89].

Recently it has been also been demonstrated that a tumor vaccine called fibroblast activation protein-α (FAP) gene-engineered tumor cell-derived exosome-like nanovesicles (eNVs-FAP) can target both tumor parenchyma and the stromal cells (CAFs) which contribute to tumor growth, metastasis, immunosuppression, and drug resistance [142]. It was shown that the eNVs-FAP vaccine effectively inhibited tumor growth in mouse models of lung cancer. The vaccine achieved this by inducing strong and specific immune responses mediated by cytotoxic T lymphocytes (CTLs) against tumor cells and FAP + CAFs [142].

## Regulation epithelial-mesenchymal transition (EMT) by TEXs in lung cancer

Epithelial-mesenchymal transition (EMT) is a critical stage and important mechanism in the progression of lung cancer metastasis [143]. In this stage, tumor cells lose the ability to adhere to epithelial cells by reducing the expression of epithelial markers like E-cadherin and occludins and by overexpressing mesenchymal markers like vimentin, N-cadherin, and alpha-smooth muscle actin (α-SMA), which gives them the ability to migrate and invade [144]. Exosomes have been shown to have a role in EMT in lung cancer [99, 145]. These exosomes convey mesenchymal-induced signals from CAFs and drive tumor cells toward a more aggressive phenotype. High quantities of vimentin are found in TEXs isolated from the serum of patients with advanced lung cancer, and these TEXs can trigger EMT in recipient human bronchial epithelial cells [145]. Exosome miRNAs have an impact on lung cancer metastasis and carcinogenesis, and exosomes derived from lung cancer cell lines have heightened miR-499a-5p levels [146]. In lung cancer, tumor-derived exosomal miR-499a-5p induces EMT via the mammalian target of the rapamycin (mTOR) signaling pathway and has both therapeutic and diagnostic potential [147]. Recent studies have shown that mesenchymal stem cells (BMSCs) generated from bone marrow are crucial to EMT, and exosomes originating from BMSCs facilitate the transfer of miR-193a-3p and miR-210-3p via activation of STAT3 signaling which, in turn, promotes cancer cell infiltration and EMT [148]. Exosomes containing the miRNA-210 are also secreted by cancer-associated fibroblasts and are picked up by lung cancer cells, where they induce cell migration, proliferation, invasion capabilities and EMT [149]. Finally, during TGF-1-mediated EMT, A549 cells release exosomes that have altered cargo in terms of protein and miRNA content. These exosomes trigger further phenotypic alterations through autocrine signaling [150]. These findings point towards the possibility that exosomal transcription factors, mRNAs and miRNAs promote lung cancer cell invasion, penetration and metastasis by acting as mediators of EMT.

## Modulation of angiogenesis by TEXs in lung TME

Angiogenesis, also known as neovascularization, refers to the process of forming new blood vessels from existing ones in the surrounding tissue. It plays a crucial role in tumor growth and metastasis, as it provides the necessary blood supply to tumors, delivering essential nutrients and oxygen for their survival and growth [151]. Tumors, including cancerous ones, require a dedicated blood supply in order to grow beyond a certain size. As a tumor grows, it requires increased amounts of oxygen and nutrients to support its metabolic needs. A delicate equilibrium between pro- and anti-angiogenic factors in the surrounding tissue closely controls the process of angiogenesis. However, pro-angiogenic proteins, which encourage the growth of new blood vessels, predominate in malignancies [32, 152]. Endothelial cells are stimulated to proliferate and migrate by pro-angiogenic factors released by tumor cells and other cells in the tumor milieu. These endothelial cells develop new blood vessels that expand in the direction of the growth, feeding it the nutrition and oxygen it requires [152]. The freshly formed blood vessels integrate into the tumor's structure as it grows, supplying a steady flow of blood and enabling tumor growth [32, 151]. The whole process is controlled by several processes, including angiogenic molecules, vascular endothelial growth factor (VEGF) and transforming growth factor beta (TGF-β) [153]. It has been shown that TEXs carry a variety of chemicals, including miRNAs which, when ingested by endothelial cells, can trigger neo-angiogenesis [32]. Exosomes produced by lung cancer are more abundant in hypoxic environments and are essential for angiogenesis [41, 154]. For instance, one study demonstrated that lung cancer cells cultured in hypoxic environments produce exosomal miR-23a, which promotes angiogenesis by inhibiting prolyl hydroxylases 1 and 2 when internalized in endothelial cells, resulting in an increase of the hypoxia-inducible factor-1. (HIF-1) [83].

Additionally, these increase vascular permeability and cancer cell migration by suppressing protein ZO-1 (Zonula occludens 1 protein), a tight junction protein [83]. An increase in angiogenesis is caused by the accumulation of miR-210 in exosomes caused by the overexpression of the tissue inhibitor of metalloproteinase-1 (TIMP-1) [155]. In addition to playing a role as a target for exosomal miRNAs in the process of promoting angiogenesis, the STAT3 signaling pathway also enhances the release of miRNAs [156]. Due to their significant influence on angiogenesis, exosomal microRNAs have been researched as potential therapeutic targets in NSCLC. For instance, exosomal miR-497 has been found to effectively decrease the production of VEGF-A, thereby inhibiting tumor development, suggesting its potential to be used as a therapeutic tool in the treatment of lung cancer [157]. Taken together, these findings highlight TEX's significance in driving tumor angiogenesis.

## Diagnostic, prognostic, and therapeutic applications of TEXs in lung cancer

Despite substantial breakthroughs in detection and therapy, lung cancer control remains a global concern. This is due to a lack of accurate biomarkers leading to ineffective early detection. Biomarkers are indications of a certain physiological or biological state in the body and are critical in medicine for diagnosing normal and abnormal states and assessing therapy response because they can be used to predict the likelihood of progression, recurrence and the efficacy of therapy in cancer settings [35, 36, 70, 158]. Exosomes may successfully replace a variety of physiologically important components, including proteins and RNA transcripts, since the cargo they transport preserves the properties of their original cells. These payloads, present in various physiological fluids, are shielded from oxidation during transit by the exosomal membrane [53].

TEXs and their constituents in bio fluids, carrying the same information as their parent cells, may enable the creation of a distinct molecular profile of a tumor which could aid in the development of non-invasive biomarkers for diagnosis, prognosis and drug resistance, which could be vital in managing patients with lung cancer. Accumulating evidence has demonstrated that analysis of plasma TEXs provide a highly sensitive approach to analyzing lung cancer, and exosomal cargoes that are altered in the tumor can be used as biomarkers for diagnosing, predicting and determining the outcome of lung cancer [41, 66, 159].

## Tumor-derived exosomal proteins in lung cancer

TEX proteins are emerging and promising cancer biomarkers [68, 160]. They are widely dispersed, have high permeability making them easily accessible and are shielded from degradation by the distinct lipid bilayer [53, 161]. TEXs are also more likely to include specific cancer-associated proteins than conventional tumor biomarkers, which improves their ability to correctly predict cancer development [161].

Several studies have researched the possibility of employing a panel of exosomal proteins for identifying lung cancer [7, 66, 111, 159, 162–166] (Table 2). Yamashita et al. investigated the efficacy of assessing EGFR expression on exosomal membranes for lung cancer and discovered considerably greater levels of exosomal EGFR expression in cancer cases than in normal controls [166]. Similarly, Huang et al. found that EGFR was present in the majority of exosomes obtained from lung cancer biopsy samples but only in approximately 2% of samples from chronic lung inflammation cases [111]. When these exosomes were co-cultured with Th0 cells, they generated regulatory T cells (Tregs) specific to tumor antigens, capable of inhibiting tumor antigen-specific CD8 + T cells. This implies that exosomes containing EGFR may play a role in promoting immunological tolerance in lung cancer, which has important implications for immunotherapy approaches.

**Table 2:** Studies on the clinical relevance of exosomal-derived miRNA and protein biomarkers in lung cancer

TEX Cargoes	Source of TEXs	Isolation and characterization	Applications	Subjects	Comment	References
EGFR	Lung Tissue biopsy	Ultracentrifugation and Flow cytometry western blotting	Therapy monitoring and diagnosis	30 NSCLC + 10 with chronic lung inflammation	Approximately 80% of exosomes isolated from LC biopsies contained EGFR. These purified exosomes suppressed CD8 + T cells specific to tumor antigens by regulatory T cells suggesting that EGFR-containing exosomes may promote immunological tolerance in lung cancer, which has ramifications for immunotherapy methods	[111]
NY-ESO-1, EGFR, PLAP, and Alix	Plasma	Extracellular Vesicle Array	Prognosis	276 NSCLC	NY-ESO-1 was linked to a lower overall survival rate, indicating its potential as a robust prognostic biomarker in NSCLC	[159]
CD151, CD171, Tetraspanin 8	Plasma	Extracellular Vesicle Array	Diagnosis	336 LC + 126 C	Exosomal proteins CD151, CD171, and tetraspanin- 8 exhibited the greatest capacity to differentiate between cancer patients with varying histological subtypes, indicating that profiling of exosome proteins has the potential to be a dependable diagnostic method for lung cancer, regardless of the subtype or stage of the disease	[66]
CDL5, C20ORF3, CLEC3B, SAA4, SERFINC1, and LSERFINF1	Serum	Polyethylene glycol-based precipitation and immunoaffinity separation- and western blot	Diagnosis	20 AC + 20 SCLC + 20 SCC + 20 C	Expression of exosomal CD5L was observed to correlate with the expression of cancer tissue, implying that it may function as a potential biomarker for the non-invasive diagnosis of lung cancer through liquid biopsy	[162]
LRG-1	Urine	Ultracentrifugation and transmission electron microscopy	Diagnosis	8 NSCLS + 10 C	LRG1 mRNA was elevated in NSCLC patients' urine exosomes and lung tissue, suggesting its promise as a diagnostic for non-invasive NSCLC detection	[7]
Panel array of 30 protein	Plasma	Extracellular Vesicle Array	Diagnosis	109 NSCLC + 110 C	Using EV Array analysis, exosomes were detected and characterized in all samples. Through multivariate analysis using the Random Forests method, a model consisting of 30 markers was generated, which successfully distinguished the NSCLC patients with controls	[4]
PD-L1	Plasma	Ultracentrifugation	Diagnosis and prognosis	51 NSCLC	In patients with advanced NSCLC, the increased expression of PD-L1 mRNA, exoPD-L1, or both during the initial stage of ICIs treatment may serve as potential indicators of positive biomarkers for enhanced effectiveness and overall survival	[167]
TIM-3 Galectin-9	Plasma	precipitation, western blotting and TEM	Diagnosis	103 NSCLC 56 C	Tim-3 and Galectin-9 levels were considerably higher in the plasma of NSCLC patients than in controls. Moreover, they were positively associated with various malignant characteristics such as larger tumor size, advanced stages, and distant metastasis, suggesting their potential use as biomarkers for NSCLC	[166]
miR-30e-3p, miR-361-5p, miR-10b-5p miR-181-5p, miR-30a-3p, miR-15b-5p, and miR-320b	Plasma	Ultracentrifugation	Early diagnosis and histological classification of different cancer types	**Set 1**. 16AC + 10SSC + 12C **Set2** 10AC + 10SSC + 30C Symptomatic = 60	Next-generation sequencing revealed the presence of adenocarcinoma-specific miRNAs and SCC-specific miRNAs in TEXs suggesting their potential as effective and sensitive biomarkers for early NSCLC detection without invasive procedures	[168]
hsa-miR-210-5p hsa-miR-9-3p, hsa-miR-205-5p,, and hsa-miR-1269a	Serum	Precipitation	Diagnosis and prognosis	147 NSCLC + 149 C	NSCLC patients had higher levels of specific micro-RNAs in serum exosomes. The study also found that miR-1269a promotes proliferation, migration and invasion in NSCLC cells	[169]
miR-378a, miR-379, miR-139-5p, and miR-200b-5p miR-151a-5p, miR-30a-3p, miR-200b-5p, miR-629, miR-100, and miR-154-3p	Plasma	Precipitation	Diagnosis	10AC + 10LG + 10C	The miR-378a, miR-379, miR-139-5p and miR-200b-5p varied significantly between lung adenocarcinomas and carcinomas compared to non-nodule samples obtained from healthy former smokers. On the other hand, miR-151a-5p, miR-30a-3p, miR-200b-5p, miR-629, miR-100 and miR-154-3p could distinguish between lung adenocarcinoma and granuloma based on their expression levels	[170]
miR-378i, miR-205-5p, and miR-200b	Pleural effusion	Ultracentrifugation	Diagnosis and histological classification	9 lung cancer + 9 pneumonia + 9 tuberculosis	The study found that miR-378i, miR-205-5p and miR-200b were expressed differently in the samples analyzed. Specifically, these miRNAs were differentially expressed in lung cancer samples, while 27 other miRNAs were differentially expressed between pneumonia and tuberculosis groups. Of note, miR-205-5p and miR-200b were significantly increased only in the lung cancer group	[171]
miR-182 and miR-210	Pleural effusion	Precipitation	Diagnosis	41 AC + 15 benign pleural effusion	The expression of miR-182 and miR-210 was significantly greater in lung adenocarcinoma samples compared to malignant pleural effusion samples. These miRNAs may serve as biomarkers to differentiate between lung adenocarcinoma and malignant pleural effusion	[172]
miR-200 and LCN2	pleural effusions	Precipitation RNA profiling	Differential diagnosis	18 AC + 18 benign inflammatory processes	Analysis of miRNA carried by exosomes in pleural effusions can potentially differentiate individuals with benign conditions from those with lung adenocarcinoma. miR-200 microRNAs and LCN2 could be useful diagnostic markers for detecting lung cancer through liquid biopsies	[173]
miR-21 and miR-4257	Plasma	Ultracentrifugation and transmission electron microscopy	Histological classification and prognosis	6 NSCLC + 129 stage 1 + 34 stage II + 32 Stage III + 30 C	Patients with non-small cell lung cancer who had higher amounts of miR-21 and miR-4257 in their plasma exosomes had a lower risk of dying from their illness. These miRNAs may one day be used as an indicator that can forecast whether or not NSCLC recurrence will occur	[174]
miR-23b-3p, miR-10b-5p and miR-21-5p	Plasma	Precipitation and Transmission electron microscopy, western blot	Diagnosis and prognosis	10 AC + 10 C	Elevated levels of miR-23b-3p, miR-10b-5p, and miR-21-5p were linked to poorer overall survival outcomes, according to independent studies of exosomes. The use of plasma exosomal miR-23b-3p, miR-10b-5p, and miR-21-5p may be a reliable non-invasive way to forecast the prognosis of NSCLC patients	[175]
miR-20b-5p and miR-3187-5p	Serum	Ultracentrifugation	Diagnosis and prognosis	330 NSCLC + 312 C	miR-20b-5p and miR-3187-5p present in circulating serum exosomes are potential candidates as NSCLC biomarkers	[176]
let-7a-5p and BCL2L1	Serum	Ultracentrifugation	Diagnosis and prognosis	154 LC + 54 pneumoconiosis + 100C	The reduced exosomal let-7a-5p expression and increased BCL2L1 expression in patients with lung adenocarcinoma can be predictive biomarkers for poor survival outcomes	[177]
miR-382	Serum	Precipitation	Diagnosis	126 NSCLC + 60 C	NSCLC patients showed a significant reduction in circulating exosomal miR-382 levels, which increased after one month of surgical resection. The decreased levels of circulating exosomal miR-382 were linked with unfavorable clinical outcomes and lower overall survival rates. Serum exosomal miR-382 was identified as an independent prognostic factor for overall survival in cases of NSCLC	[178]
eIF4E	Serum	Precipitation and western blotting	Prognosis	99 NSCLC + 40 C	Higher levels of eIF4E in NSCLC tissues were associated with advanced stages of the disease and poorer overall survival rates. In the NSCLC group, exosomal eIF4E expression in the serum of individuals with advanced TNM stage, distant metastasis, and CYFRA 21–1 was significantly higher compared to that of healthy individuals	[179]
miR-146a-5p	Serum	Precipitation and Transmission electron microscopy	Diagnosis	100 NSCLC	The exosomal miR-146a-5p found in the serum has the potential to act as a new biomarker for anticipating the efficacy of cisplatin treatment in patients with NSCLC. Furthermore, it can also be utilized for the continuous monitoring of drug resistance in real-time	[180]
miR-1169 and miR-260	Plasma		Diagnosis		Exosomal miR-1169 and miR-260 have been identified as potential biomarkers with distinct properties that can differentiate between NSCLC patients with wild-type EGFR and those with mutant EGFR during the early stages of the disease	[181]

*Abbreviations***:** *AC* adenocarcinoma, *CYFRA21-1* cytokeratin 19 fragment; *CEA* carcinoembryonic antigen, *CYFRA 21–1* serum-positive cytokeratin fragment 19, *C* controls, *eIF4E* Eukaryotic translation initiation factor 4E, *EGFR* Epidermal growth factor receptor, *EV* extracellular vesicles, *HR* hazard ratio, *IP* interstitial pneumonia, *LCN2* Lipocalin 2, *LC* lung cancer, *NSCLC* non-small cell lung cancer, *NTA* nanoparticle tracking analysis, *P* pneumonia, *PFS* progression-free survival, *SCC* squamous carcinoma, *SCLC* small cell lung cancer, *T* tuberculosis, *TEM* transmission electron microscopy, *TNM* tumor-node-metastasis, *WB* western blot

Sandfeld-Paulsen et al. investigated the diagnostic accuracy of a set of membrane-attached exosomal proteins in 336 lung cancer patients and 127 control participants using an array that allows multiplex analysis of several exosomal proteins [159]. They found that CD151, CD171 and tetraspanin 8 were the most effective biomarkers for distinguishing between those with and without lung cancer, as well as differentiating between various types of lung cancer histology [159]. Furthermore, they discovered that exosomal proteins obtained from plasma could potentially forecast the prognosis of NSCLC patients in another study to phenotype exosomes from plasma of NSCLC patients capturing 49 proteins [66]. The study reported a notable association between placental alkaline phosphatase (PLAP), ALG-2-interacting protein X (ALIX), the CTA New York Esophageal Squamous Cell Carcinoma-1(NY-ESO-1), and EGFR, with a decreased overall survival rate emphasizing the potential advantages of utilizing exosomal membrane-bound proteins as robust prognostic biomarkers in NSCLC [66].

Another study identified potential lung cancer biomarkers by profiling extracellular vesicle-derived proteins in healthy individuals and cancer patients using proteomic analysis and differential expression of arrays of proteins [162]. Among these proteins, CD5L was identified as a promising biomarker with high accuracy for diagnosing lung cancer and was also found to be associated with various lung cancer histologies. Urine exosomal biomarkers were also found to assist NSCLC diagnosis in a proteomic study by Li et al. [7]. They reported that patients who were initially suspected of having lung cancer but were later found to be cancer-free had elevated levels of leucine-rich alpha-2-glycoprotein (LRG1) in urine-derived exosomes when compared to controls indicating that LRG1 obtained from urine might be a feasible non-invasive biomarker for diagnosing NSCLC [7]. Another study by Feng Wu et al. demonstrated that exosomes obtained from bronchoalveolar lavage (BAL) fluid of both smokers and NSCLC patients contained higher levels of certain proteins, such as human leukocyte antigens -class I (HLA-class I), B melanoma antigen (BAGE), PD-L1, and annexin-A2, compared to healthy individuals [165].

Furthermore, the study also revealed that the expression of exosomal miRNAs, mRNAs, and lncRNAs differed between smokers and NSCLC patients, indicating that smoking might affect the exosome's capacity to regulate molecular components within exosomes and could be a contributing factor to the onset of lung cancer [165]. Further, Wang et al. reported that lipopolysaccharide-binding protein (LBP) levels in exosomes obtained from the serum of NSCLC patients were considerably higher than in healthy individuals [164]. Notably, the expression of exosomal LBP was higher in patients with metastatic NSCLC than those without metastasis, suggesting exosomal LBP may serve as a potential biomarker for metastasis and distinguish between patients with metastatic and non-metastatic NSCLC [164]. Gao et al. found that plasma-derived exosomes from NSCLC patients expressed T-cell immunoglobulin and mucin domain 3 (Tim-3) and galectin-9 in much higher quantities than those of healthy individuals (103 NSCLC patients and 56 healthy individuals) [163]. Moreover, they reported that the exosomal expression of Tim-3 and galectin-9 exhibited a positive correlation with various clinico-pathological features such as patient age, tumor size, distant metastasis, and cancer stage, suggesting them as potential biomarkers for NSCLC [163].

## Tumor-derived exosomal miRNA in lung cancer

Exosomal miRNAs are very small non-coding RNA molecules that have been linked to the etiology of lung cancer [168]. Cancer cells release these miRNAs into the extracellular region, where they are transported in exosomes through the bloodstream. Exosomal miRNAs can control gene expression by binding to target mRNA molecules and either blocking or causing their degradation once they reach their target cells [169].

Several investigations have found that exosomal miRNAs play a role in lung cancer progression via a variety of pathways [148, 157, 168, 169]. Exosomal miRNAs, for example, have been shown to support angiogenesis, a critical step in tumor development, by causing endothelial cell proliferation and migration [33, 170]. Exosomal miRNAs have also been linked to the control of vascular permeability, which can promote cancer cell invasion and spread [31, 33]. Furthermore, exosomal miRNAs have the potential to be useful indicators for early lung cancer detection. Most of these circulating exosomal miRNAs have been evaluated as biomarkers, mainly in diagnosis and prognosis. In most cases, the clinical utility does not rely on a specific miRNA but on a panel of multiple miRNAs [171].

In a recent study, Jin and colleagues explored the discriminatory ability of TEX biomarkers between squamous cell carcinoma and adenocarcinoma in patients with initial-stage NSCLC by exosomal miRNA profiling in plasma samples [172]. They reported a number of exosomal miRNAs, including those specific to adenocarcinomas which could be used as non-invasive biomarkers for early NSCLC diagnosis. Another study by Wang et al. demonstrated that a panel of four miRNAs (hsa-miR-9-3p, hsa-miR-205-5p, hsa-miR-210-5p and hsa-miR-1269a) detected in serum-derived exosomes might be utilized to identify NSCLC patients [173]. These miRNAs were more abundant in NSCLC patients than in healthy people and could distinguish between the two groups, and of the four miRNAs, miR-1269 displayed the highest discriminatory ability [173]. Cazzoli et al. examined 742 miRNAs in a study involving 30 individuals with lung adeno-carcinomas, lung granulomas, and healthy smokers. They discovered a diagnostic panel of miR-379, miR-378a, miR-200b-5p, and miR-139-5p that may be used as an indicator for the diagnosis of lung cancer [174].

Notably, lung cancer patients may also be differently diagnosed by exosomal RNA profiling from pleural effusions. Lin et al. conducted a study to investigate whether analyzing the exosomal profile in pleural effusions could help in the early detection of lung cancer [175]. They analyzed the miRNA expression patterns in pleural effusions from patients with lung cancer, pulmonary TB, and pneumonia. They identified 27 miRNAs that were differentially expressed between the study groups. Notably, they found that two specific miRNAs, miR-205-5p and miR-200b, were expressed at significantly higher levels in the lung cancer samples compared to the pneumonia samples suggesting that these miRNAs could be used as potential markers to distinguish between lung cancer and pneumonia [175].

Tamiya and colleagues conducted exosomal miRNA profiling in patients with lung adenocarcinoma associated with malignant pleural effusion and reported increased levels of miR-182 and miR-210 [176]. These microRNAs could potentially be utilized as a diagnostic tool to distinguish lung adenocarcinoma pleural effusion from benign pleural effusion. Moreover, Hydbring et al. [177] suggested that microRNAs that are differentially expressed, including those belonging to the miR-200 family, have greater diagnostic precision in individuals with lung adenocarcinoma than those with pleural effusions induced by noncancerous lung diseases.

Studying the prognostic capacity of miR-21 and miR-4257 in a large cohort of 195 NSCLC patients and 30 healthy controls, Dejima et al. [178] found that, plasma levels of exosomal miR-21 and miR-4257 from NSCLC patients were markedly upregulated in patients with recurrence than those without recurrence and were associated with a poorer prognosis and a shorter disease-free life. Moreover, miR-21 was associated with clinical factors such as the size of the tumor and its stage of metastasis to the nodule, while miR-4257 was associated with histological type and TNM stage [178]. Another study has reported elevated levels of exosomal miR-10b-5p, miR-23b-3p, and miR-21-5p independently associated with poor overall survival in NSCLC patients [179]. Additionally, circulating serum exosomal miR-20b-5p and miR-3187-5p levels are significantly lower in patients with early-stage NSCLC compared to healthy individuals [180].

Moreover, Zhang and colleagues [181] revealed that the downregulation of blood-derived exosomal let-7a-5p was significantly linked to the advancement of lung adenocarcinoma and poor survival in individuals exposed to dust at work. Another study reported that significantly decreased levels of exosomal miR-382 in NSCLC patients and was positively associated with poor clinical variables, including overall survival [182]. Dong et al. [183] demonstrated the serum-derived exosomal-eIF4E in NSCLC individuals to be notably higher compared to healthy individuals suggesting that augmented levels of exosomal eIF4E in NSCLC tissues were associated with advanced TNM stage, distant metastasis and late-stage disease, consequently leading to poor survival.

Yuwen et al. [184] investigated the impact of tumor-derived exosomal miR-146a-5p levels on NSCLC chemo- to cisplatin and the molecular process through which it influences chemotherapy responsiveness. They observed that NSCLC patients with advanced illness who had low serum- derived exosomal miR-146a-5p levels were more likely to recur than those with heightened levels, suggesting that serum exosomal miR-146a-5p might be a useful biomarker for predicting cisplatin effectiveness and monitoring treatment resistance in NSCLC patients [184]. Furthermore, differentially expressed exosomal miRNAs in serum have recently been identified as potentially useful prediction indicators for EGFR mutations in NSCLC [185]. They reported miR-1169 and miR-260 to be possible biomarkers which are able to discriminate between initial-stage NSCLC caused by wild-type and mutant EGFR [181].

## Tumor-derived exosomal long noncoding RNAs (lncRNAs) in lung cancer

Long non-coding RNAs (lncRNAs) are a class of RNA molecules that differs from protein-coding mRNAs in that they do not encode proteins, and are transcribed from DNA and can range in length from over 200 nucleotides to more than 100,000 nucleotides, [186]. Despite being non-coding, lncRNAs play important regulatory roles in gene expression and can interact with DNA, RNA, and proteins to modulate various cellular processes [186]. They regulate important biological processes such as cell differentiation, cell division and epigenetic regulation [187]. Researchers have discovered that lncRNAs are dysregulated and expressed abnormally in various tumors, including lung cancer, and more recently, that they influence tumor development, metastasis and invasion in exosomes secreted by lung cancer cells [188].

One of the most intensively researched possible biomarkers for detecting NSCLC is metastasis-associated lung adenocarcinoma transcript 1(MALAT1), which accelerates tumor migration and proliferation by suppressing cell apoptosis shortening the cell cycle when highly expressed in NSCLC patient serum [189]. A recent study has demonstrated a substantial accumulation of lncRNA- SOX2 overlapping transcript (lncRNA-SOX2OT) in exosomes obtained from the peripheral blood of NSCLC patients with bone metastases [190]. Furthermore, patients displaying elevated levels of exosomal lncRNA-SOX2OT exhibited significantly shorter overall survival rates, suggesting it could be a promising target for the treatment of metastatic NSCLC [190]. Notably, lncRNA-SOX2OT overlapping transcript and ANRIL may be the ideal biomarkers for predicting the prognosis of NSCLC because these have been found to be elevated in tissues and serum samples, compared to healthy controls and decreased levels are related to greater overall survival rates [191].

Other potential markers of NSCLC metastasis include six urinary exosomal-derived lncRNAs lnc-(SRY-11, lnc-FRAT1-5, and lnc-RNASE13-1), which exhibited significantly higher expression levels in NSCLC patients compared to healthy individuals. Conversely, lnc-ARL6IP6-4, lnc-RP11-80A15.1.1–2, and lnc-DGKQ-1 expression levels were considerably down-regulated in NSCLC patients [67]. Also, exosomal linc01125, might be a novel and reliable biomarker for diagnosing NSCLC, predicting prognosis and assessing survival rates because of its ability to distinguish NSCLC cases from disease-free and tuberculosis patients and its association with unfavorable overall survival [192].

It has been reported that NSCLC patients exhibit significant upregulation of serum exosomal small nucleolar RNA Host Gene 15 (lncRNA SNHG15) expression compared to individuals with benign lung lesions or without the disease. These upregulated expressions of lncRNA small Nucleolar RNA Host Gene 15 (SNHG15) were identified as an independent predictor of overall survival and closely associated with the differentiation of NSCLC patients across all stages compared to controls [193]. Another study reported that NSCLC patients had significantly elevated levels of lncRNAs-actin filament-associated protein 1 antisense RNA 1 (lncRNA AFAP1-AS1) expression compared to individuals without the disease [194]. More recently, exosomal LncRNA RP5-977B1 has been found to be significantly elevated in NSCLC compared to healthy controls [195]. In a validation study, NSCLC patients showed higher levels of exosomal long non-coding RNAs TGF-β induced LncRNA (TBILA) and AGAP2 antisense RNA 1 (AGAP2-AS1) than healthy individuals as well as a significant positive association between the levels of these exosomal lncRNAs and lymph node, tumor size, and TNM stage [196].

Despite the encouraging results from the studies on exosomal-derived lncRNAs as possible candidate biomarkers for lung cancer mentioned earlier, the majority of research on lncRNAs is still in the preclinical phase, and there is a lack of understanding about their mechanism of action. Therefore, there is a pressing need for more comprehensive studies on lncRNAs to enable the effective development of cancer diagnosis and treatment strategies.

## TEXs in the targeted drug resistance therapy of lung cancer

Drug resistance is a significant obstacle that hinders the effectiveness of chemotherapy, radiotherapy, and targeted treatment. Despite a strong initial response to these therapies, most patients with NSCLC acquire drug resistance within 9 to 12 months [197]. As a result, improving clinical outcomes for NSCLC patients require better knowledge of the molecular processes behind treatment resistance and the discovery of predictive biomarkers for targeted therapy. Drug resistance develops when drug-sensitive tumor cells undergo intracellular pathway alterations or activate paracrine and autocrine pathways that aid survival. In response to various treatments, these cells also express a wide range of molecules [198]. Exosomes, essential for cell-to-cell communication, have been associated with developing drug resistance in cancer [199, 200]. Exosomal proteins have gained recognition as significant mediators of drug resistance. Exosomes can precisely deliver functional P-glycoprotein to drug-sensitive recipient cells. This transfer initiates signaling pathways that are essential for the development of drug resistance in these cells [201]. This phenomenon has been seen in NSCLC, where cisplatin resistance has been noted as a prevalent side effect of the intravenous administration of this platinum-based DNA-damaging medication.

It has been demonstrated that hypoxia worsens drug resistance in lung cancer cells by increasing the expression of pyruvate kinase isozymes M2 (PKM2) in NSCLC. An upregulation had been observed in exosomes released by hypoxic cisplatin-resistant cells. Hypoxic conditions contribute to drug resistance in lung cancer cells through various mechanisms. Firstly, NSCLC cells increase their glycolysis, producing metabolites that counteract the reactive oxygen species triggered by cisplatin. Secondly, hypoxia-induced changes in CAFs create an acidic microenvironment that promotes NSCLC cell proliferation and resistance to cisplatin. These findings shed light on the complex relationship between hypoxia, exosomes, and the development of drug resistance in lung cancer cells [202].

A growing body of evidence indicates that TEXs play a role in drug resistance to EGFR- Tyrosine kinase inhibitors (EGFR-TKIs) through the transfer of exosomal miRNAs [21, 203]. Exosomes released by EGFR-TKI-resistant cells decrease the susceptibility of NSCLC cells to gefitinib, and this resistance may be overcome by blocking miR-21 [21]. Additionally, exosomes containing a secondary T790M mutation of EGFR have been found to induce drug resistance in EGFR-TKI-sensitive cells and are associated with increased expression of exosomal miR-3648 and miR-522-3p [204]. Similarly, exosomal miR-214 inhibition can overcome medication resistance caused by miR-214 overexpression in gefitinib-resistant cells [205].

TEXs have also been implicated in anaplastic lymphoma kinase (ALK)-TKI resistance, where exosomes from resistant subclones induce resistance in originally sensitive sub-clones through differential expression of miRNAs and lncRNAs. A recent study has shown that exosomes produced by lung cancer cells facilitate the spread of cisplatin resistance to other cancer cells. This resistance was associated with a decrease in the levels of miR-100-5p, which consequently resulted in the downregulation of mTOR expression [206]. In addition, miR-206 has a role in the control of cisplatin resistance as well as the EMT process that occurs in human lung cancer cells [207]. Specific RNA molecules in serum exosomes can predict how well patients with NSCLC respond to cisplatin therapy. These RNA molecules may serve as biomarkers for detecting drug resistance in real-time. miR-146a-5p expression steadily declines in both NSCLC cells and the exosomes they release as drug resistance arises in response to cisplatin therapy. The fundamental mechanism is that miR-146a-5p targets Atg12 and inhibits autophagy, increasing the susceptibility of NSCLC cells to cisplatin [184].

**Fig. 3:**
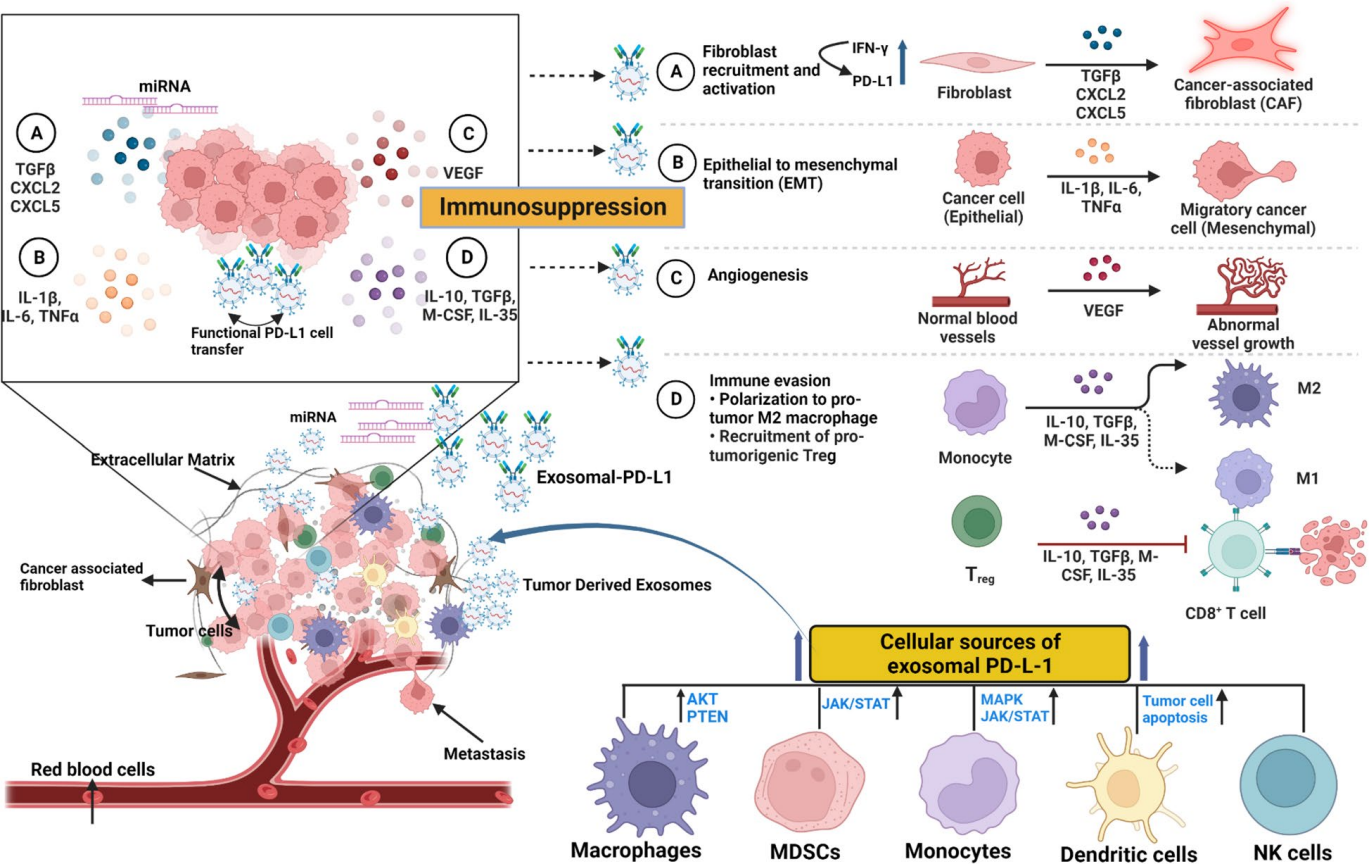
Mechanisms of exosome-induced drug resistance in lung cancer: **A** Exosomes, derived from CAFs and drug-resistant tumor cells, can be efficiently internalized by drug-sensitive cells. Subsequently, the contents of these exosomes, including nucleic acids, miRNA, lncRNA, circRNA and proteins, play a significant role in transmitting the drug-resistance phenotype to the drug-sensitive cells. Cancer cells secrete exosomes that transfer miRNAs to fibroblasts in the TME, leading to the differentiation of CAFs and conferring drug resistance in cancer cells by stimulating metastasis and proliferation while suppressing the pro-apoptotic function of FOXO3a and activating the mTOR/PTEN/P13K/ATK signaling pathway. This activation is directly linked to the inhibition of apoptosis and tumor progression, effectively preventing drug-induced apoptosis. Furthermore, exosomes can enhance drug resistance in sensitive cells by transferring ABC transporters, which are responsible for actively expelling drugs out of the cell. **B** Therapeutic exosomes can be loaded with therapeutic drugs and miRNAs in vitro. Exosomes loaded with drugs can be taken up by tumor cells, directly causing their death by altering the transcriptome and TME, affecting growth, proliferation, angiogenesis, promoting EMT, and even impacting drug resistance. Moreover, the surface markers of these exosomes can be modified in vitro to enhance their ability to target tumors. Exosomes can also serve as stimulators for DC vaccines, promoting immune responses. Exosomes expressing tumor-associated antigens can stimulate DCs, enabling them to activate T cells, ultimately leading to the destruction of tumor cells. Figure created with BioRender.com

Moreover, recent investigations have highlighted the role of lncRNAs and exosome-mediated pathways in fostering lung cancer treatment resistance. Tumor-derived lncRNA H19 has been linked to gefitinib resistance [208]. According to a study by Yu et al., exosomes released by NSCLC cells resistant to the EGFR-TKI icotinib showed increased expression of the oncogene MET in exosomes isolated from metastatic NSCLC patients [209]. These findings highlight the significance of TEXs in mediating drug resistance in lung cancer. The possible mechanism by which exosomes regulate the drug resistance in TME in lung cancer is depicted in Fig. 3.

## TEXs as promising drug delivery vehicles and cancer vaccines in targeted lung cancer therapy

Tumor-derived exosomes are getting recognition as promising and very efficient delivery systems for drugs used in the therapy of cancer. They are being explored widely as a prospective alternative due to their inherent role in intercellular communication, high biocompatibility, low immunogenicity, ability to remain in the blood circulation for longer periods, biodegradable qualities, and capacity to transverse through various biological barriers [15, 210, 211]. Over the past few years, exosomes have emerged as promising carriers for delivering drugs in cancer treatment [167, 212–215]. A list of studies employing therapeutic approaches that rely on exosome targated drug/ and non-coding RNA delivery systems as a modality for lung cancer therapy/ treatment is outlined in Table 3. Engineered exosomes, derived primarily from dendritic cells, mesenchymal stem cells, macrophages, or cancer cells, are utilized for encapsulating tumor-targeting therapeutic drugs such as RNAs or proteins [216]. These exosomes are chosen as they can exhibit tumor-associated antigens and modulate immune responses [217]. The process involves mixing chemotherapy drugs or RNAs with exosomes, followed by encapsulation through electroporation or sonication [217, 218].

**Table 3:** Summary of studies outlining using exosomes as targeted drug / and non-coding RNA delivery as a modality for Lung cancer therapy/ treatment

Sources of Exosomes	Loading drugs /Non-coding RNA	Target cells	Outcome / Therapeutic Effects	References
Bovine Milk	Withaferin A, Curcumin, Paclitaxel (PTX) and docetaxel (DTX)	A549, H1299 cancer cells, human normal bronchial epithelial cells (Beas-2B)	Enhanced tumor targetability and tumor reduction by milk-derived drug-loaded exosomes	[219]
Bovine Milk	Paclitaxel (PTX)	A549 lung cancer cells	Augments Antitumor efficacy. Exosomal PTX administered orally exhibited significant tumor growth inhibition against human lung tumor xenografts in mice	[220]
Bovine Milk	Celastrol (CEL)	A549, H1299 NSCLC cells	Enhanced anti-tumor efficacy in NSCLC. Exo-CEL exhibited enhanced anti-tumor efficacy and less toxicity as compared to free CEL, both in vitro and in vivo	[221]
Bovine Milk	si-KRASG12S	A549 lung cancer cells	Dose-dependent anti-proliferative by exosomal siKRASG12S in A549 cells. Significant inhibition of A549 tumor xenografts treated with folic acid-functionalized exosomes carrying siKRAS in vivo and in vitro	[222]
A549 tumor cells derived exosomes	Docetaxel	A549 lung cancer cells	Exo-DTX effectively inhibited A549 cell growth, enhanced cytotoxicity, induced apoptosis, increased ROS production, and demonstrated anti-cancer effects in vitro. In vivo, experiments showed that Exo-DTX could serve as a targeted treatment option with superior drug potency compared to free DTX	[223]
macrophage-derived exosomes (RAW 264.7)	Paclitaxel	Murine Lewis lung carcinoma cell subline (3LL-M27 cells)	Vectorized exosomes, loaded with PTX, exhibited a significant loading capacity and demonstrated a remarkable ability to accumulate in lung cancer cells after systemic administration resulting in improved therapeutic outcomes	[224]
macrophage-derived exosomes) Raw 264.7	Paclitaxel	Cancer cells (MDCKMDR1, MDCKwt, and and (3LL-M27)	Exo-PTX significantly increased cytotoxicity in drug-resistant MDCKMDR1 (Pgp +) cells. The administration of exoPTX resulted in a potent anticancer effect and a high degree of co-localization with cancer cells mouse model of Lewis lung carcinoma pulmonary metastases	[225]
Bovine colostrum-derived exosomes	Paclitaxel	A549 lung cancer cells	ExoPTX exhibited enhanced anti-proliferative activity compared to PAC alone against A549 cells and drug-resistant variants of A549 cells. The oral dose of ExoPAC attached to tumor-targeting ligand folic acid (FA-Exo-PTX) was more effective in inhibiting tumor development in the orthotopic lung cancer model than conventional intravenous administration of PTX	[226]
MDA-MB-231 cells	MiRNA-126	A549 lung cancer cells	miRNA-231-Exo loaded with miRNA-126 inhibited lung cancer cell proliferation and metastasis in mice	[227]
Engineered Exosomes HEK293T	siRNA	A549 lung cancer cells	Targeting siRNA-tLyp-1 exosomes effectively silences cancer cell genes and decreases cancer stem cells' stemness. These engineered exosomes demonstrated high transfection efficiency, making them a promising gene delivery platform for future gene cancer therapy	[228]
Engineered exosomes with PD L-1 antibody	PD-L1siRNA	A549 and H460 lung cancer cells	In vitro, Inhibition of tumor cell proliferation suggests a potential for siRNA as efficient delivery carriers for tumor immunotherapy and gene therapy	[229]
A549 cells (Lung cancer cells derived exosomes	miR-563	A549 lung cancer cells	MiR-563 suppressed the expression of Bcl-2, an anti-apoptotic protein, in A549 cells. This led to increased apoptosis (programmed cell death) in the cells, which resulted in tumor regression and improved survival in vivo	[230]
Engineered exosomes (tLyp-1-modifed EVs) HEK293T cells	SOX-2-siR1, siR2, siR3	A549 lung cancer cells	Engineered exosomes Inhibited cancer cell growth by Knocking down the expression of the SOX2 gene and decreased the stemness of cancer stem cells in NSCLC	[231]
Human cell-derived exosomes HEK293T cells	miRNA-497	A549 lung cancer cells	Human cell-derived exosomes as carriers for miRNAs cultured in both 2D and 3D microfluidic devices revealed that exosomes loaded with miRNA-497 (miR-497) had potent anti-tumor and anti-angiogenic effects, which effectively inhibited tumor growth and suppressing the expression of associated genes in A549 cells	[157]

A recent study has shown that engineered lung-specific exosomes (231-Exo) loaded with miRNA-126 evaded immune surveillance. They effectively hindered the growth and movement of A549 lung cancer cells via disruption of phosphatase and tensin homolog/phosphatidylinositol 3-kinase/protein kinaseB (PTEN/PI3K/AKT) signaling pathway. Furthermore, in a mouse model with lung metastasis, the miRNA-231-Exo exhibited targeted delivery to the lungs and notably suppressed the formation of lung metastases [227]. Soluble FMS-like tyrosine kinase 1 (sFlt-1) has been shown to have anti-tumor effects via inhibiting angiogenesis in a number of cancer models [232]. Engineered TEXs have been loaded with sFlt-1 to take advantage of this therapeutic potential. This therapeutic formulation has shown promising anti-tumor action, resulting in increased tumor apoptosis and suppression of tumor cell proliferation. Furthermore, it has demonstrated superior efficacy in inhibiting pro-angiogenic processes [232].

Similarly, it has been shown that exosomes, coupled with the anti-cancer drug paclitaxel and engineered to incorporate the aminoethylanisamide-polyethylene glycol vector, can specifically target the sigma receptor that is overexpressed in lung cancer cells. This innovative approach led to a substantial accumulation of engineered exosomal complexes within cancer cells after systemic delivery and a better therapeutic outcome [224].

Developing a cancer vaccine represents an innovative and forward-thinking scientific strategy to address global health crises associated with cancer. Exosomes may be used in cancer therapy because they include multivalent surface portions from living cells that are impossible to mimic in artificial nanoparticles [233]. Exosomes from immune cells, cancer cells, and healthy cells have all been compared for their efficacy in the treatment of cancer [212, 234].

Exosomes have recently been found to have great promise for cancer immunotherapy, positioning them as a promising tool in developing effective cancer vaccines and an optimal platform for advancing the development of next-generation cancer vaccines [235]. Because they contain natural tumor antigens that can efficiently activate APCs, TEXs, are being modified to create cancer vaccines. Many studies have documented the beneficial effects of TEX-based vaccination, including T-cell mediated anti-tumor immune responses and a diminution of tumors [70–72, 236, 237]. For instance, in melanoma animal models, TEX vaccination has protected against tumor development and inhibited lung metastasis. At the same time, in syngeneic mice, exosomes produced by L1210 leukemia cells have restricted tumor growth and given resistance to the tumor [237, 238]. Research indicates that exosomes derived from DCs can trigger T-cell-mediated immune responses against cancer, and numerous clinical trials are currently underway to explore the application of educated dendritic cells in the treatment of lung cancer [235]. Recently, it has been shown that the delivery of miRNA-30a through exosomes has effectively hindered lung cancer metastasis [235].

A relatively untapped field of exosome research is the study of exosomes generated from plants. Exosomes may be able to exhibit anticancer effects and avoid problems from possible post-cancer therapies, according to ongoing research in this area [212]. Moreover, TEXs are unlikely to produce enough anti-tumor immunity because they contribute to immunosuppression and have low immunogenicity. To increase antigen immunogenicity, a number of techniques are being developed to create efficient and affordable treatments based on exosomes. These include the use of electroporated siRNA, engineering TEXs to express both tumor-associated and pathogenic antigens, affixing well-known immune boosters like CpG DNA and TLR ligands, directly fusing TEXs with antigens, and using external stimuli to increase TEX release [55, 212, 239].

Though the TEX-based exosomal vaccine's initial results appear promising, novel strategies are needed to make TEX-based cancer vaccines a reality. Future therapies might be made possible by engineering TEXs to activate their anti-tumor potential. Understanding the signals inside TEX cargo and connecting them with clinical data is now the biggest challenge in finding and verifying tumor biomarkers. However, implementing TEXs might completely transform how cancer is diagnosed and treated.

## Roles of TEXs immune checkpoint proteins in lung cancer

Immune checkpoints are signaling molecules produced by immunological cells and are thought to be guardians of immune responses [58]. In lung cancer, these pathways play a complex role in promoting and inhibiting the ability of the immune system to fight cancer. Over the past several decades, a number of immune checkpoints have been identified, and numerous studies have shown their contribution to the development of tumors by enhancing anti-tumor immune responses in lung cancer [240]. The treatment of advanced NSCLC has been drastically transformed by ICIs, which can be administered alone or in conjunction with chemotherapy [241]. Recent research has uncovered the expression of different immunological checkpoint proteins, including programmed death ligand-1 (PD-L1), cytotoxic T-lymphocyte associated protein 4 (CTLA-4), and TIM-3, in TEXs [242–244]. More and more studies suggest that exosomal immunological checkpoint proteins are involved in the regulation of tumor immune evasion by a novel mechanism and may serve as novel targets for cancer immunotherapy. [245–247].

## Tumor-derived PD-L1 and immunotherapy in lung cancer

Programmed death-ligand 1 (PD-L1) is a type I transmembrane protein found primarily on the surface of cancer cells. It can attach to the PD-1 receptor on T cells, inhibiting T cell activation and supporting immunological homeostasis [248]. The interaction of PD-L1 and PD-1 on T cells has been shown to inhibit T cell activation, proliferation, and cytokine release, resulting in a weakened immune response against cancer cells. This immune checkpoint signaling pathway is critical in tumor cell immune escape, allowing them to avoid immunological detection and elimination by the host immune system [249].

**Fig. 4:**
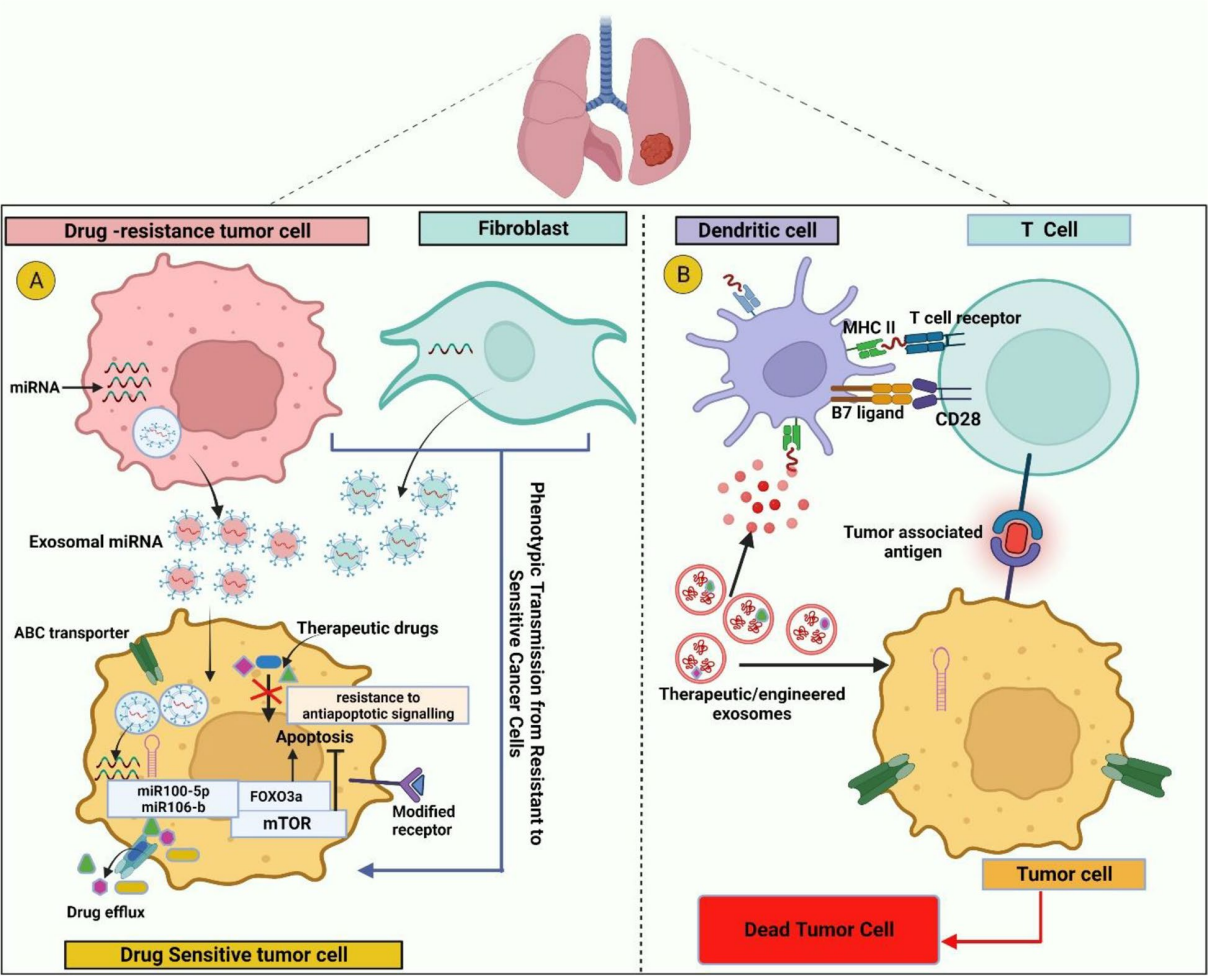
Overview of cellular crosstalk driven by tumor derived exosomes and exosomal-PD-L1 in the tumor microenvironment: The cellular components of lung TME include immune cells, fibroblasts and epithelial cells with extracellular matrix and blood vessels. Among the many processes linked to the hallmarks of lung cancer, such as immune evasion, the establishment of a metastatic environment and angiogenesis, cancer cells secrete chemicals that regulate the TME and contribute to the progression of cancer. Exosomal PD-L1 (exo-PD-L1) is found not only in cancer cells but also in other immune cells, such as dendritic cells, macrophages, monocytes, natural killer cells, MDSCs.Other cells may also be sources of exo-PD-L1 which mediates functional PD-L1 transfer between cells and induces systemic immunosuppression microenvironments to facilitate metastasis. Tumor-derived exosomes (TEXs) are responsible for inducing PD-L1 expression in immune cells. This transpires through the upregulation of AKT/PTEN, MAPK and JAK/STAT signaling by regulatory proteins and microRNA. Cancer-associated fibroblasts (CAF) also express PD-L1 in response to interferon-γ (IFN-γ), a key activator of macrophages, natural killer cells and neutrophils. TEXs cause fibroblast activation (**A**), which leads to the secretion of soluble compounds that enhance PD-L1 expression in tumors and cause epithelial to mesenchymal transition phenotype (**B**). Similar to this, TEXs induce the PD-L1 to collaborate via vascular endothelial growth factor (VGEF) and alter angiogenesis (**C**) to sustain TME. The immune evasion is facilitated by pro-tumorigenic T regulatory (T reg) (D). Exosomal PD-L1 inhibits T cell activation by stimulating the production of CD8 + cytotoxic T lymphocytes, often known as CTLs. These lymphocytes target tumor cells and trigger apoptosis through their cytotoxic activities, a cytotoxic effect of CD8 + T lymphocytes which can be inhibited by exo-PD-L1. High concentrations of PD-L1 in exosomes can lead to apoptosis in activated CD8 + T lymphocytes. Figure created with BioRender.com

Tumor cells have elevated levels of PD-L1 expression which can shield them from immune monitoring by T cells by attaching to PD-1 on activated T cells [249]. When PD-L1 and PD-1 bind together, they can suppress the activity of T cells, making it harder for the immune system to attack cancer [250]. In addition to the cell surface of many tumor cell types, PD-L1 is also found on exosome surfaces called exosomal PD-L1 (exo-PD-L1) [59]. An expanding body of evidence suggests that TEXs with the PD-L1 protein on their surface play a role in angiogenesis, tumor formation, infiltration, metastasis and immune evasion [55]. Studies have confirmed that the interplay between exo-PD-L1 and activated immune cells drive a tumor's immunosuppressive mechanism [251–253]. Exo-PD-L1 is more robust and resistant to proteolytic enzyme degradation and may also have more potent immunomodulatory effects in the bloodstream and TME [254]. PD-L1 is expressed in the TME by various cell types, including macrophages, DCs, MDSCs and tumor cells and exo PD-L1 may also originate from these cell types and may be transmitted to different cell types, such as tumor cells, macrophages and DCs [248, 250]. In general, the actions of exo-PD-L1 regulate T cells and control other immune cells in order to produce the milieu of the tumor immuno-suppressive and prevent an anti-tumor immune response. In Fig. 4 the cellular crosstalk driven by TEXs and exosomal-PD-L1 in the TME involving its transfer and upregulation is illustrated.

Clinically, blocking PD-L1 with an antibody activates the anti-tumor immune response, resulting in prolonged remission in certain cancer patients [56, 249]. However, the majority of individuals had adaptive resistance [56, 255]. Notably, utilizing anti-PD-L1 antibodies to block exo-PD-L1 may enhance anti-tumor immune response and, more effectively, reduce tumor development. As a result, exo-PD-L1 offers a unique therapeutic approach to address the problem of antibody resistance shown in existing techniques.

Until recently, limited research has been conducted on the potential role of PD-L1 expression in TEXs as a biomarker for cancer patients who receive ICIs. Several investigations [60, 62, 163, 256–258] have reported a substantial increase in the expression of immunological checkpoint proteins in TEXs in lung cancer patients. Zang et al. investigated the correlation between exo-PD-L1 and immunohistochemistry (IHC) PD-L1 status and the pathological characteristics of NSCLC patients undergoing anti-PD-L1 therapy [259]. They reported a correlation between exo-PD-L1 levels and IHC PD-L1 status and exo-PD-L1 levels and lymph node metastasis, indicating that their identification might forecast the effectiveness of ICIs treatment in lung cancer patients [259]. A further study analyzing the clinical importance of PD-L1 expression in serum-derived exosomes from a sample of 85 patients with NSCLC found exoPD-L1 expression associated with tumor size, lymph node status, metastasis and NSCLC progression [256]. Another study exploring the prognostic significance of exoPD-L1 and CD28 in NSCLC patients receiving ICIs demonstrated that patients with elevated levels of exo-PD-L1 and decreased levels of CD28 had shorter progression-free survivals implying that aggregate baseline levels of exoPD-L1 and CD28 could be of predictive significance of anti-PD1 therapy [57].

Although recent research has discovered that exo-PD-L1 is associated with lung cancer prognosis, there are still some caveats. First, it is difficult to discern between the pharmacologic effect of the PD-1 inhibitor and the efficacy of autoimmunity during PD-1 therapy. Second, the bulk of studies focus on a retrospective cohort with a relatively small sample size and is therefore prospective; large-scale clinical trials are still necessitated to corroborate these results. Third, a clear methodology for dealing with exo-PD-L1 due to tumor cell heterogeneity is yet to be established.

Nevertheless, Exo-PD-L1 is a broader paradigm innovation with promising therapeutic and further research applications. Exo-PD-L1 suppresses immunity, even though the precise mechanism by which it does so is poorly understood. Future research should focus on the assays determining how exo-PD-L1 affects different populations of immune cells (such as NK cells, DCs, macrophages, Tregs and B cells), the mechanism behind the impact of induced exo-PD-L1 expression on cancer progression and whether patients develop an autoimmune response to exo-PD-L1. The complexity of the tumor-immune microenvironment implies that more complete models should be utilized in assessing immunotherapy instead of the traditional method of pinpointing genes to identify an appropriate biomarker.

## Future perspectives, challenges, and conclusion

The current review highlights TME immunomodulatory properties of exosomes produced from tumor cells. It is clear that, TEXs are important intercellular communication mediators in healthy and pathological conditions. They alter oncogenic pathways in cancer cells and play a significant mechanistic role in the tumor microenvironment, all of which have been shown to promote tumor growth, metastasis, and resistance to treatment of lung cancer.

Due to their nonoscale dimensions and capacity for ferrying molecules into certain recipient cells, TEXs have been proposed as potential drug delivery systems, which makes them especially intriguing for biological applications like biomarker molecules and anticancer vaccines.

Exosomes can be used as non-invasive biomarkers to complement or supplement standard biopsy since the tumor exosomal payload contains chemicals from the releasing cells and can be found in the blood. Investigating the intricate make-up of exosomes can result in precise, swift medical interventions, screening and detecting initial-stage lung cancer for more favorable prognoses and developing a multianalyte strategy with the potential to offer dynamic insight into the tumor microenvironment.

Nevertheless, exosome analysis has yet to be incorporated into clinical recommendations due to numerous hurdles yet to be overcome before exosome-based diagnosis/prognosis and delivery systems can be applied in clinical settings. The primary and most significant obstacle is related to the isolation and comprehensive characterization of exosomes. The lack of uniform procedures for isolating exosomes, appropriate quality controls, and storage methods have hindered the development of medical-grade exosome manufacturing and limited analysis in standard clinical laboratories. Secondly, exosome analysis would benefit from economical and simple-to-use specialized technology. Exosome heterogeneity is intimately associated with the various activities of exosome subgroups with diverse molecular profiles. As a result, single-particle assays which can discriminate between exosome biological origins, size, content and functional influence on recipient cells, could be used to identify exosomes.

Furthermore, considerable evidence from in vitro and in vivo animal studies support the involvement of TEX in regulating an immunosuppressive milieu for tumor development. Nevertheless, the relatively short time frame of these and other preclinical investigations may not reflect the dynamic process of cancer cell immunogenicity determined by the phenotype of the encompassing microenvironment and throughout which additional immune evasion mechanisms may evolve. This problem is exacerbated by the paucity of clinical trial research evaluating the therapeutic potential of TEXs in lung cancer and the fact that most studies are retrospective with small cohorts. Consequently, additional prospective studies with larger populations are required to prove TEX use as a liquid biopsy and credible alternative to tumor tissue biopsy.

Thereby, the selection of exosome indicators which best correspond with clinical state from among various studies are complicated. Whenever these challenges are resolved, exosomes will most likely play an important role in lung cancer treatment. To do so, further translational research and clinical trials must be conducted before incorporating exosomes into lung cancer treatment.

Overall, the available data from preclinical analyses of TEX molecular cargo and their effects on different immune cells support the critical role of TEX in establishing an immunomodulatory microenvironment, which may influence various cancer activities such as invasion, metastasis, EMT and angiogenesis. Notwithstanding the drawbacks and difficulties, a thorough comprehension of the TEXs molecular profile and complex interactions with immune cells in a tumor microenvironment may result in efficient, customized immunotherapy which enhances therapeutic outcomes.

Exosome administration of therapeutic drugs is a revolutionary strategy with a bright future in medicine because of the distinct biological properties of exosomes. Medical care for lung cancer should be accurate and individualized, and we propose that TEXs may be altered to improve clinical outcomes and enhance clinical lung cancer care. More research efforts at various levels are required to accomplish this.

## Data Availability

Not applicable.

## Abbreviation

EMT: Epithelial-mesenchymal transition
SCLC: Small-cell lung cancer
NSCLC: Non-small cell lung cancer
TME: Tumor microenvironment
ICIs: Immune checkpoint inhibitors
EVs: Extracellular vesicle
MVBs: Multivesicular bodies
TEXs: Tumor-derived exosomes
ESCRT: Endosomal sorting complex required for transport
ECM: Extracellular matrix
CAFs: Cancer-associated fibroblasts
NKs: Natural killer cells
DCs: Dendritic cells
MDSCs: Myeloid-derived suppressor cells
TNF: Tumor necrosis factor-α
